# Benchmarking feature projection methods in radiomics

**DOI:** 10.1038/s41598-025-16070-w

**Published:** 2025-09-05

**Authors:** Aydin Demircioğlu

**Affiliations:** Institute of Diagnostic and Interventional Radiology and Neuroradiology, University Hospital Essen, Hufelandstraße 55, 45147 Essen, Germany

**Keywords:** Feature projection, Feature selection, Feature reduction, Interpretability, Radiomics, Machine learning, Biomarkers, Medical research

## Abstract

In radiomics, feature selection methods are primarily used to eliminate redundant features and identify relevant ones. Feature projection methods, such as principal component analysis (PCA), are often avoided due to concerns that recombining features may compromise interpretability. However, since most radiomic features lack inherent semantic meaning, prioritizing interpretability over predictive performance may not be justified. This study investigates whether feature projection methods can improve predictive performance compared to feature selection, as measured by the area under the receiver operating characteristic curve (AUC), the area under the precision-recall curve (AUPRC), and the F1, F0.5 and F2 scores. Models were trained on a large collection of 50 binary classification radiomic datasets derived from CT and MRI of various organs and representing different clinical outcomes. Evaluation was performed using nested, stratified 5-fold cross-validation with 10 repeats. Nine feature projection methods, including PCA, Kernel PCA, and Non-Negative Matrix Factorization (NMF), were compared to nine selection methods, such as Minimum Redundancy Maximum Relevance (MRMRe), Extremely Randomized Trees (ET), and LASSO, using four classifiers. The results showed that selection methods, particularly ET, MRMRe, Boruta, and LASSO, achieved the highest overall performance. Importantly, performance varied considerably across datasets, and some projection methods, such as NMF, occasionally outperformed all selection methods on individual datasets, indicating their potential utility. However, the average difference between selection methods and projection methods across all datasets was negligible and statistically insignificant, suggesting that both perform similarly based solely on methodological considerations. These findings support the notion that, in a typical radiomics study, selection methods should remain the primary approach but also emphasize the importance of considering projection methods in order to achieve the highest performance.

**Supplementary Information:**

The online version contains supplementary material available at 10.1038/s41598-025-16070-w.

## Introduction

Radiomics is a quantitative approach that leverages image analysis and machine learning techniques to analyze radiological image data^1–4^. By extracting numerous morphological, intensity and textural features, radiomics transforms medical images into quantitative data that captures the underlying patterns and variations^3,5^. This data is then used to create models for diagnostic, prognostic or therapeutic decision making^6–9^.

Central to radiomics is the establishment of a link between quantitative features and biological biomarkers, enabling a non-invasive approach to personalized medicine^10^. However, since it is unknown which feature may correspond to a biomarker, radiomics typically involves the extraction of a large number of features^11^. In addition, imaging preprocessing filters like smoothing, sharpening etc. are often applied to further enhance the expressiveness of the extracted features^12,13^. Both result in features that are highly correlated and potentially model noise. Such features are problematic, because they can lead to overfitting and can reduce the interpretability of the resulting model. Removing them will generally improve model accuracy, and will also result in computational gains^14,15^.

To remove redundant and irrelevant features, the vast majority of radiomic studies currently rely on feature selection methods, ranging from simple techniques such as t-Tests to more advanced approaches such as Minimum Redundancy Maximum Relevance (MRMR)^16,17^. These methods aim to identify features that are strongly associated with and are predictive of the outcome. In contrast, feature projection methods, such as Principal Component Analysis (PCA)^18^take a different approach by generating new features by recombining the original ones. These methods aim to retain as much information as possible in a non-redundant manner, and their focus is therefore not on predictive power but on creating features that represent the data more efficiently. However, this recombination can compromise interpretability. For example, while the volume of a lesion is inherently interpretable, a feature derived from the sum of its volume and the entropy of its texture is not easily understood by humans. As a result, feature projection methods are rarely used in radiomics^19^where the primary goal is to establish interpretable links between extracted features and biomarkers.

Nevertheless, the notion that radiomic features are interpretable and can be directly linked to biomarkers is not without its critics^20,21^. While morphological features such as volume and sphericity have clear, interpretable meanings, the majority of radiomic features, particularly textural features, are more abstract and not easily understood by humans^22–24^. In fact, radiomics was developed specifically to capture features that are beyond human perception or qualitative description^2^. This challenges the rationale for relying mainly on feature selection methods to preserve features as it may compromise the predictive performance of the resulting model. Currently, it remains unclear whether feature projection techniques offer advantages over feature selection, as no systematic comparison has been performed.

In this study, the gap was addressed by benchmarking a range of feature projection and feature selection methods on a large collection of radiomic datasets to evaluate their predictive power to understand whether feature projection is a viable alternative to the de facto standard of feature selection in radiomics. To assess the impact of feature reduction techniques, a rigorous nested cross-validation strategy was implemented in which a model consisting of a feature reduction method and a common machine learning classifier was trained and evaluated. Model performance was measured using the area under the receiver operating characteristic curve (AUC), area under the precision-recall curve (AUPRC), and the F1, F0.5, and F2 scores. In addition, the influence of dataset characteristics (sample size, number of features, dimensionality, and class balance) on the difference between the two techniques were measured.

## Results

**Fig. 1:**
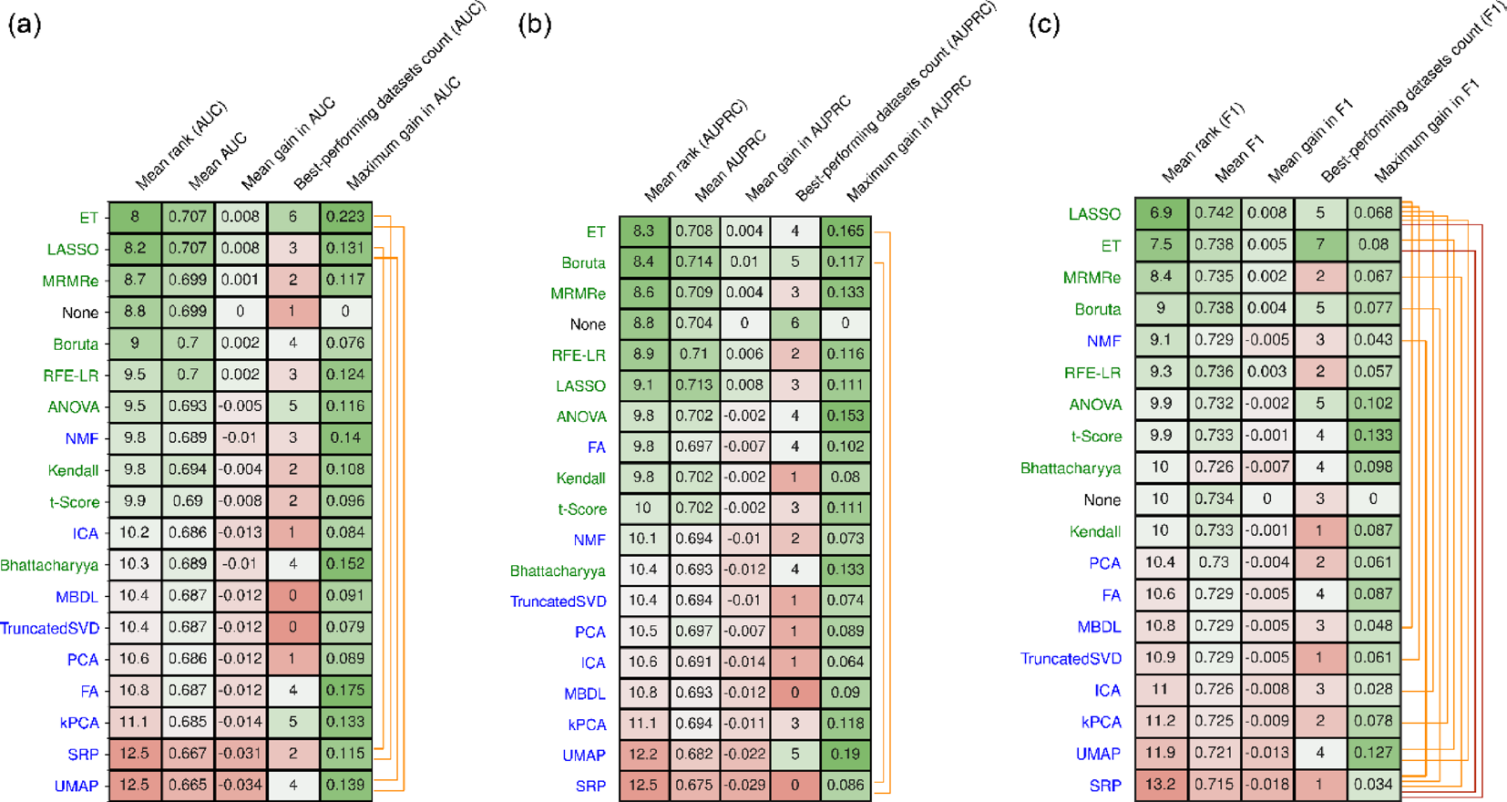
Ranking of feature reduction methods. Ranking of feature reduction methods with respect to (a) AUC, (b) AUPRC, and (c) F1 score. Methods in green represent feature selection, while those in blue represent feature projection methods. The mean rank was calculated by ranking all methods for each dataset and averaging the ranks. The mean gain was calculated as the difference in performance compared to no feature reduction (None). The number of best performing datasets indicates the number of datasets on which the corresponding method performed best. The maximum gain represents the highest performance improvement observed across all datasets compared to using no feature reduction. The methods were tested for differences using a Friedman test followed by a post-hoc Nemenyi test. Methods with significant differences are connected by an orange line (*p* < 0.05) or a red line (*p* < 0.001).

A total of 9,500 models were computed across 50 datasets, 10 repeats, and 19 methods. On average, feature selection methods emerged as the best performers (Fig. 1). Extremely Randomized Trees (ET) and Least Absolute Shrinkage and Selection Operator (LASSO) achieved the highest average AUC, with mean ranks of 8 and 8.2, respectively (Fig. 1a). However, they were ranked best on only 6 and 3 datasets, respectively. The most effective feature projection method was Non-Negative Matrix Factorization (NMF), which achieved an average rank of 9.8. Notably, NMF performed worse than not reducing the features at all, with a mean loss of AUC of 0.01. The least effective methods were Uniform Manifold Approximation and Projection (UMAP) and Supervised Random Projection (SRP), although even these methods performed best on 4 and 2 datasets, respectively. The commonly used PCA was less effective than all feature selection methods and was the best performing method on only one dataset.

Similar patterns emerged when considering AUPRC or F-scores (Fig. 1, Supplementary Materials, Figure S1). Again, feature selection generally led to better performance, with LASSO, ET, Boruta and MRMRe remaining the best-performing methods. Slight differences were visible only for the F2 score, where nearly all methods performed better than applying no feature reduction. NMF performed relatively better in this case, becoming the second-best method (Supplementary Materials, Figure S1e).

A Friedman test indicated significant differences among the methods for all metrics (*p* < 0.004). The post-hoc Nemenyi test showed that the main significant differences were between UMAP and SRP and the best-performing feature selection methods like ET or LASSO (Fig. 1, Supplementary Materials, Figure S1). Therefore, UMAP and SRP can be considered significantly inferior to the best-performing feature selection methods, regardless of the metric employed.

**Fig. 2:**
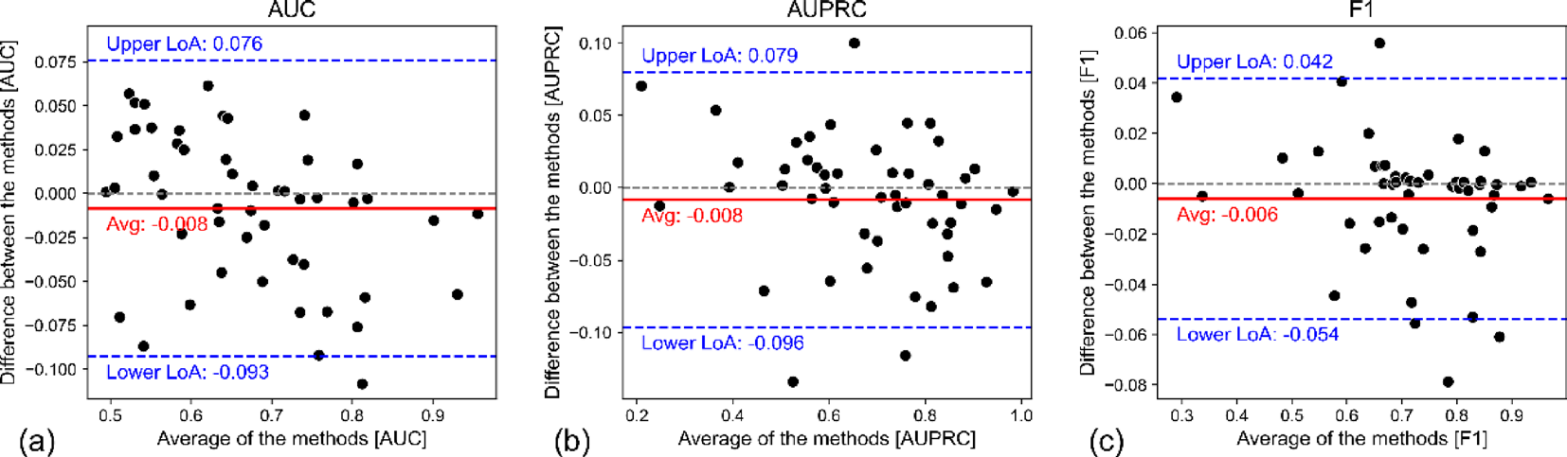
Bland-Altman plots comparing projection and selection methods. Bland-Altman plots displaying the agreement between the best-performing projection and selection methods for (a) AUC, (b) AUPRC, and (c) F1 score on each dataset. Each plot displays the difference between the two methods against their average. The solid red line indicates the mean difference, while the blue dashed lines represent the upper and lower limits of agreement (LoA), which are calculated as the mean difference ± 1.96 times the standard deviation of the differences. A positive average difference indicates that at least one feature projection method outperformed all selection methods for that dataset, whereas a negative difference indicates that at least one feature selection method outperformed all projection methods.

To examine the differences between the two methods from a methodological viewpoint, Bland-Altman analyses conducted. These indicated that, across all evaluation metrics, neither method outperformed the other, and their average difference was negligible across all evaluation metrics (Fig. 2, Supplementary Materials Figure S2). For the large majority of datasets, the differences between the methods remained within the limits of agreement (LoA), and Wilcoxon tests showed no statistical differences (all *p* > 0.15). However, the results varied widely across the datasets. For example, in terms of AUC, the best feature selection method (Boruta) outperformed the best feature projection method (PCA) by over 0.10 on WORC-LIPO, while Kernel PCA performed about 0.06 better than Bhattacharyya on Zhang2023 (Supplementary Materials, Table S1). The feature projection method that most often produced the best performing models was Factor Analysis (FA) (*N* = 9). Although SRP and UMAP performed worst on average, they outperformed the other feature projection methods on 8 and 5 datasets, respectively. For feature selection, Bhattacharyya performed best (*N* = 9), while Kendall performed relatively worst (*N* = 2). Similar patterns were observed for the other metrics as well.

**Fig. 3:**
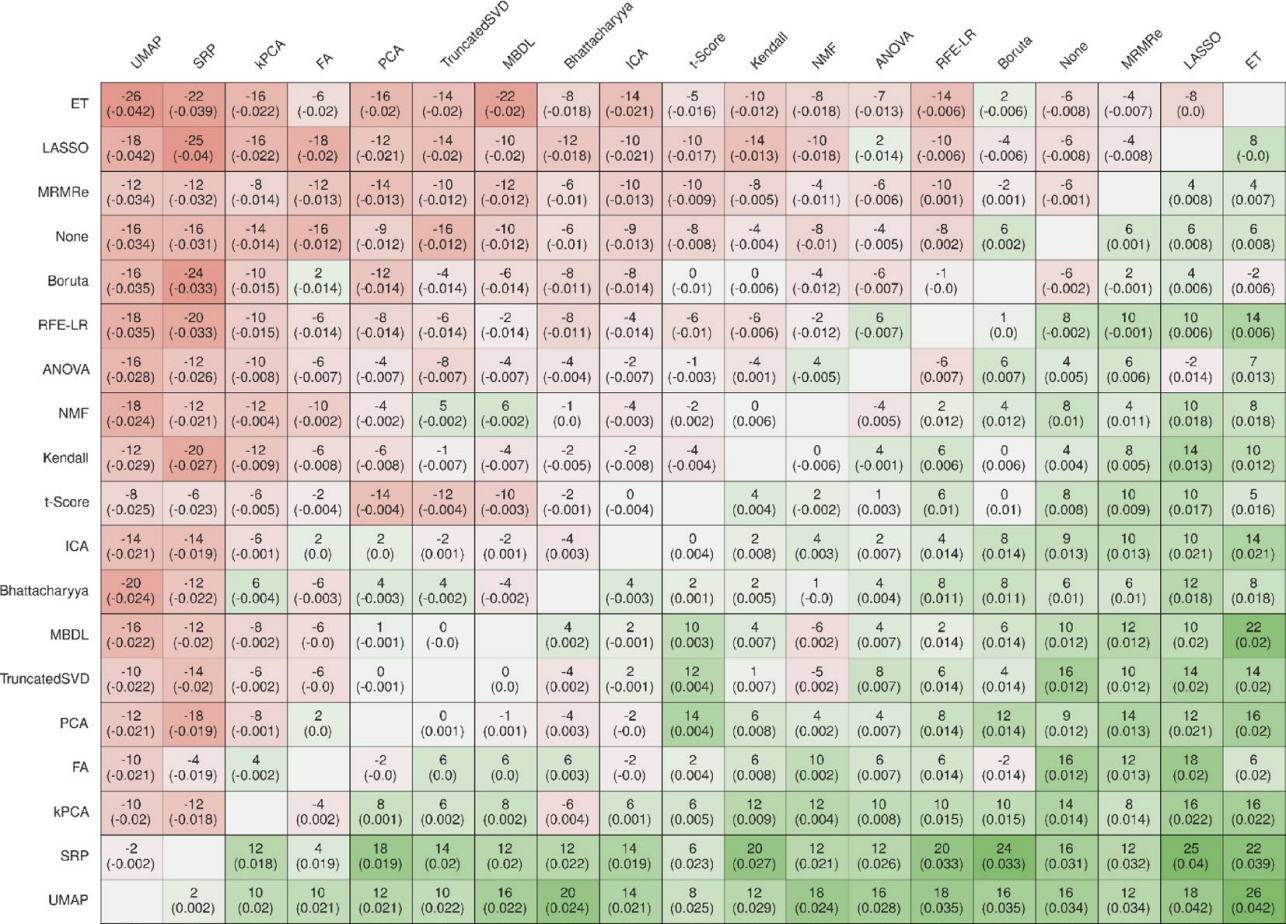
Benefit and gain comparison in AUC. The top number in each cell indicates the number of datasets, where replacing one method with another method results in a gain or loss in performance. For example, replacing ET with UMAP would lead to lower performance on 26 data sets. The number in parentheses represents the average AUC gain in this case. For instance, replacing ET with UMAP would result in an average AUC loss of 0.042 across all datasets.

Replacing one method with another showed that replacing any of the best-performing feature selection methods with feature projection does rarely lead to an overall gain in AUC (Fig. 3). These results were similarly true across all metrics (Supplementary Materials, Figure S3A-S3D). The worst performing methods, UMAP and SRP, can nearly always be improved by replacing them with any other method.

**Fig. 4:**
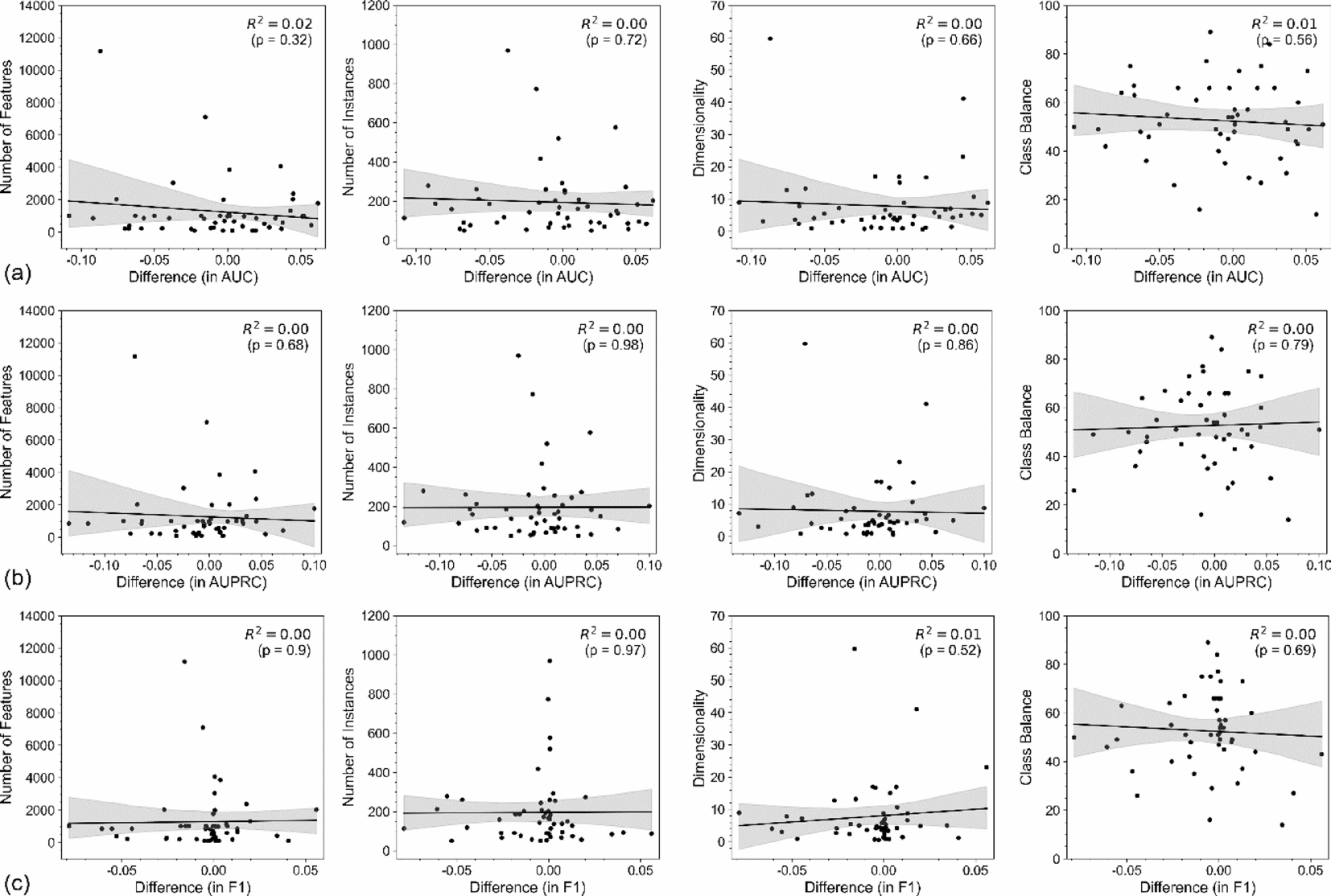
Relationship between the observed difference and dataset characteristics. Each scatter plot displays a dataset characteristic against the difference in (a) AUC, (b) AUPRC, and (c) F1 score between the best feature projection and selection methods. From left to right: number of features, number of instances, dimensionality, and class balance. The lines represent a linear regression of the corresponding variables. The R2 and p-values for the linear regression are shown in each subplot. The shaded areas represent the 95% confidence intervals for the regression predictions obtained by bootstrapping.

Testing whether the differences in AUC between the best-performing feature selection and projection methods could be attributed to dataset characteristics revealed no significant relationships (Fig. 4, Supplementary Materials, Figure S4). Neither the number of features, instances, dimensionality, nor the class balance were significantly associated with the differences between the two methods (all *p* > 0.05).

**Fig. 5:**
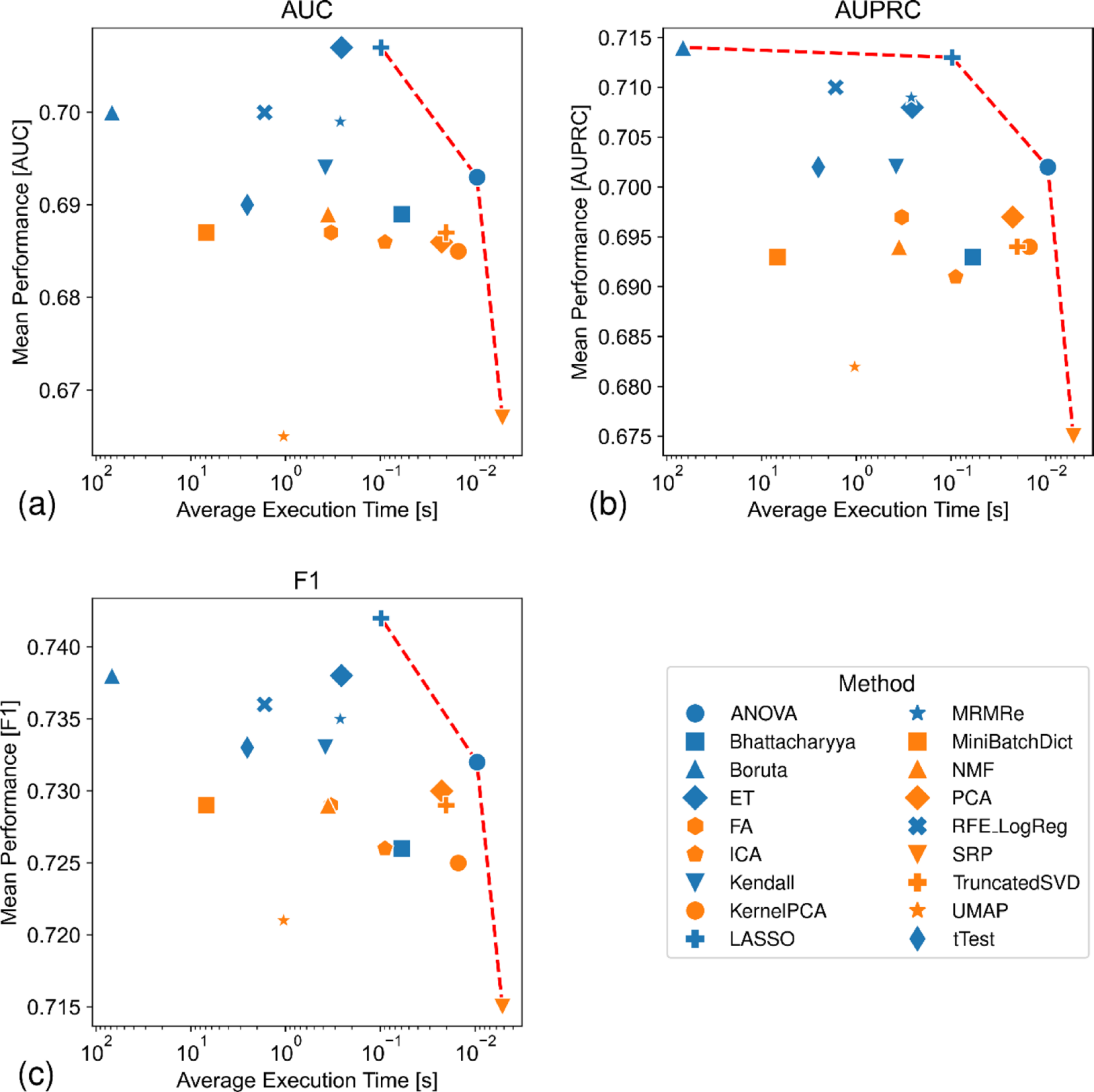
Computational efficiency. Each plot shows the average computation time (in seconds on a logarithmic scale) versus average performance across all datasets for each method. The red line indicates the Pareto front, comprising methods that are not outperformed in both speed and performance. Metrics shown: (a) AUC, (b) AUPRC, and (c) F1-score.

Regarding computational efficiency and execution times, strong differences between the methods were observed (Fig. 5 and Supplementary Materials, Figure S5 and S6). Overall, selection methods were more efficient than projection methods. LASSO was among the best performing and most efficient methods, while ANOVA performed worse but was overall faster. SRP was most often the fastest method, but exhibited the poorest performance. Most methods were not very sensitive to the number of features considered, but FA, NMF and MRMRe showed exponential behavior (Supplementary Materials, Figure S6). Unexpectedly, the computation time for applying t-Score decreased slightly as the number of features to select increased, whereas projecting to a single feature took much longer than expected when using UMAP. Mini-Batch Dictionary Learning (MBDL) and, especially, Boruta had much higher computation times than the other methods.

## Discussion

Feature selection is commonly used in radiomics to eliminate redundant and irrelevant features. In contrast, feature projection methods are less frequently applied, primarily due to concerns about potential loss of interpretability. The purpose of this study was to evaluate whether well-known feature projection methods could yield more predictive models regardless of these concerns.

The results of the analysis showed that the best performing methods on average were ExtraTrees, LASSO and Boruta, all of which are feature selection methods. The best feature projection method was NMF, while PCA, which is sometimes used in radiomics studies, performed worse than any of the feature selection methods tested. These results indicate that for all datasets, feature selection should be preferred over feature projection. Even worse, not using any feature reduction performed better on average in terms of AUC or AUPRC than the all feature projection methods, suggesting there may to be no reason to use feature projection at all.

Preferring feature selection over projection methods is only a partial reflection of the truth, since the results are highly dependent on the dataset. Although feature projection methods performed significantly worse than ET and LASSO on average, they can outperform them, sometimes even significantly, on certain datasets; for example, UMAP was the best method on 4 of the 50 datasets with respect to AUC. Despite this, the average differences between all feature selection and projection methods across all datasets were virtually nonexistent, showing that they fare similarly from a methodological point of view. Thus, in practical terms, both approaches should be evaluated if high predictive performance is the main goal for a specific dataset.

A suggestive explanation for the results is that the feature selection is supervised and therefore can use the outcome to select more predictive features. However, it is important to note that the subsequent classifiers are also supervised. Therefore, there is no compelling reason why feature reduction should not focus on reducing redundancy and noise, leaving the task of identifying relevant features to the classifier. In addition, one might have expected supervised feature projection to work best, but the only such method used, SRP, did not perform well, contrary to expectations that this approach might improve on unsupervised PCA.

Although this study suggests that the best method depends on the dataset, in radiomics feature selection offers an additional advantage. In the case that only a few features contribute, the other features do not need to be computed in new data and will not affect the resulting model. In contrast, feature projection often requires the computation of a large number, if not all, features, especially for Kernel PCA, where the nonlinear kernel makes clear attribution difficult. Since radiomic features are known to be strongly affected by reproducibility issues^21,25,26^, consequently, models using feature projection methods are likely to be more impacted.

This study considered a large collection of datasets covering various binary outcomes across different organs, which is important for obtaining a comprehensive overall insight. Considering only a few datasets may give a false impression. For example, using only two datasets, UCSF-PDGM and Arita2018, this study would have suggested that, in terms of AUC, PCA performs as well as ET for predicting IDH mutation status in glioma patients, and that PCA, which is conceptually simpler, is good enough.

Therefore, previous findings, which often rely on only a single dataset and differ in their methodologies, making comparisons unviable, must be interpreted with caution. Sun et al. evaluated glioma grading in the BraTS-2018 dataset and found that LASSO was among the top-performing methods, while PCA performed only moderately^27^. Van Gómez et al. examined seven feature selection methods in a cohort of patients with metastatic breast cancer using^18^F]-F-FDG PET/CT, reporting that LASSO performed best, with a gain of about + 0.09 in AUC over the best feature projection method^28^. Similarly, Li et al. compared LASSO and PCA in a cohort of rectal cancer patients^29^. LASSO performed slightly better for predicting response to neoadjuvant chemotherapy, but the difference became much larger (about + 0.20 in AUC) for predicting pathologic complete response. Song et al. introduced a latent representation method for subtype classification of non-small cell lung cancer (NSCLC)^30^. While LASSO achieved a slightly higher AUC than PCA, their new method performed best. Similarly, Ye et al. investigated feature projection methods to predict grade and overall survival in a cohort of patients with glioma^31^. They stated that FA is an improvement over PCA, but the difference was only + 0.02 in AUC. For feature selection methods, Lambin et al. considered their performance in a cohort of lung cancer patients, reporting that the method based on the Wilcoxon test performed best^32^. Finally, a recent benchmarking study comparing only feature selection methods found MRMRe to be best-performing, followed by LASSO and ET^16^.

Overall, the current study’s results are generally aligned with those of prior studies, particularly in highlighting the strong and consistent performance of LASSO and MRMRe across many datasets. However, the findings also support the notion that no method is universally superior^33^ and reinforces the importance of using a rigorous, unbiased evaluation framework like the nested cross-validation employed in this study.

As a consequence, it is recommended to test multiple feature selection and projection methods rigorously in radiomic studies to achieve the highest predictive performance. In addition to commonly used methods in radiomics such as LASSO, lesser-known methods such as ET and NMF should also be considered. Since the overall execution times of the methods were generally low, testing multiple methods should be feasible in many cases. Therefore, incorporating feature projection methods in future radiomic studies is advisable.

This study focused exclusively on either feature projection or selection methods. However, a mixture of both approaches may be a viable option that should be explored in future studies. One possibility could be to select interpretable features such as volume, sphericity, etc., and apply feature projection only to the vast majority of uninterpretable features, compressing them into very few features and thus leaving the resulting model partially interpretable.

Currently, radiomics places significant emphasis on interpretability^34–36^ yet many radiomic models still do not perform well enough to be considered for clinical use^37,38^. While interpretability is crucial for understanding model behavior and ensuring trust in clinical settings, achieving a model’s predictive performance that meets clinical standards should be the primary focus. Only once a model demonstrates sufficient performance should interpretability be prioritized to gain deeper insights into its workings^21^.

While the results suggest that feature projection methods could be beneficial, their broader adoption in radiomics may remain limited. Most studies continue to rely on established methodologies, particularly well-known feature selection methods like LASSO, while the use of more advanced approaches, such as those based on deep learning, remains rare^39,40^. It is unclear whether these methods will gain wider acceptance and use in the near future.

This study has limitations. First, the analysis was performed on tabular data, whereas applying feature reduction directly to images might have been of interest. In fact, feature projection methods have long been applied in image processing with varying success. However, a larger data collection of radiomic imaging is not available currently. The tabular data was also influenced by factors such as segmentation quality and software robustness, therefore, the presented results indirectly depend on these factors. Then, only datasets with high dimensionality from either CT or MRI were considered. It is unclear whether the results will hold for other imaging data, such as ultrasound or histopathological data, especially if these datasets are larger and result in datasets with low dimensionality. Additionally, the datasets considered were relatively balanced with respect to class distribution (up to 1:10). Therefore, the results might not generalize to highly imbalanced datasets. However, such extreme imbalance is uncommon in radiomics, possibly due to the typically low overall sample sizes. The datasets used might also contain unaccounted biases, such as those arising from demographic differences. Such information is often unavailable in radiomics datasets, which typically contain only imaging data. Consequently, it remains unclear how these potential biases might have affected the results.

Another limitation arises from computational constraints. Only few projection methods were considered in the present study. The main focus was on commonly used and well-performing projection methods with implementations in the sci-kit library. This restriction allowed a fair comparison, as it is likely that their implementations are comparable in quality. Regarding the feature selection methods, those that are either often used in radiomics or have performed rather well were included^16^. Additionally, the hyperparameters of these methods were not tuned. For example, UMAP involves parameters for the number of neighbors to consider, the minimum distance between two projected points, and the metric used to measure distances. While it is reasonable to expect that hyperparameter tuning could substantially improve performance, the main observation, that selection and projection methods can outperform each other depending on the dataset, is likely to remain valid. Moreover, in radiomics, hyperparameters of feature selection methods are rarely tuned in practice, which makes the present study highly relevant to current practices. A study systematically investigating hyperparameter tuning in radiomics is currently missing and should be undertaken. Finally, deep learning methods were also excluded from this study, as their training is more complex and involved.

In conclusion, feature reduction methods were investigated on a large set of radiomics datasets, and it was shown that, while feature selection methods performed better than projection, the best methods depended on the specific dataset. Therefore, to achieve the highest predictive performance, both methods should be tested in radiomic studies.

## Methods

The ethical approval for this study was waived by the local ethics committee (Ethik-Kommission, Medizinische Fakultät der Universität Duisburg-Essen, Germany) since only publicly available datasets were employed. All methods and procedures were performed following relevant guidelines and regulations. Code and data can be accessed via a GitHub repository.

### Overall study design

**Fig. 6:**
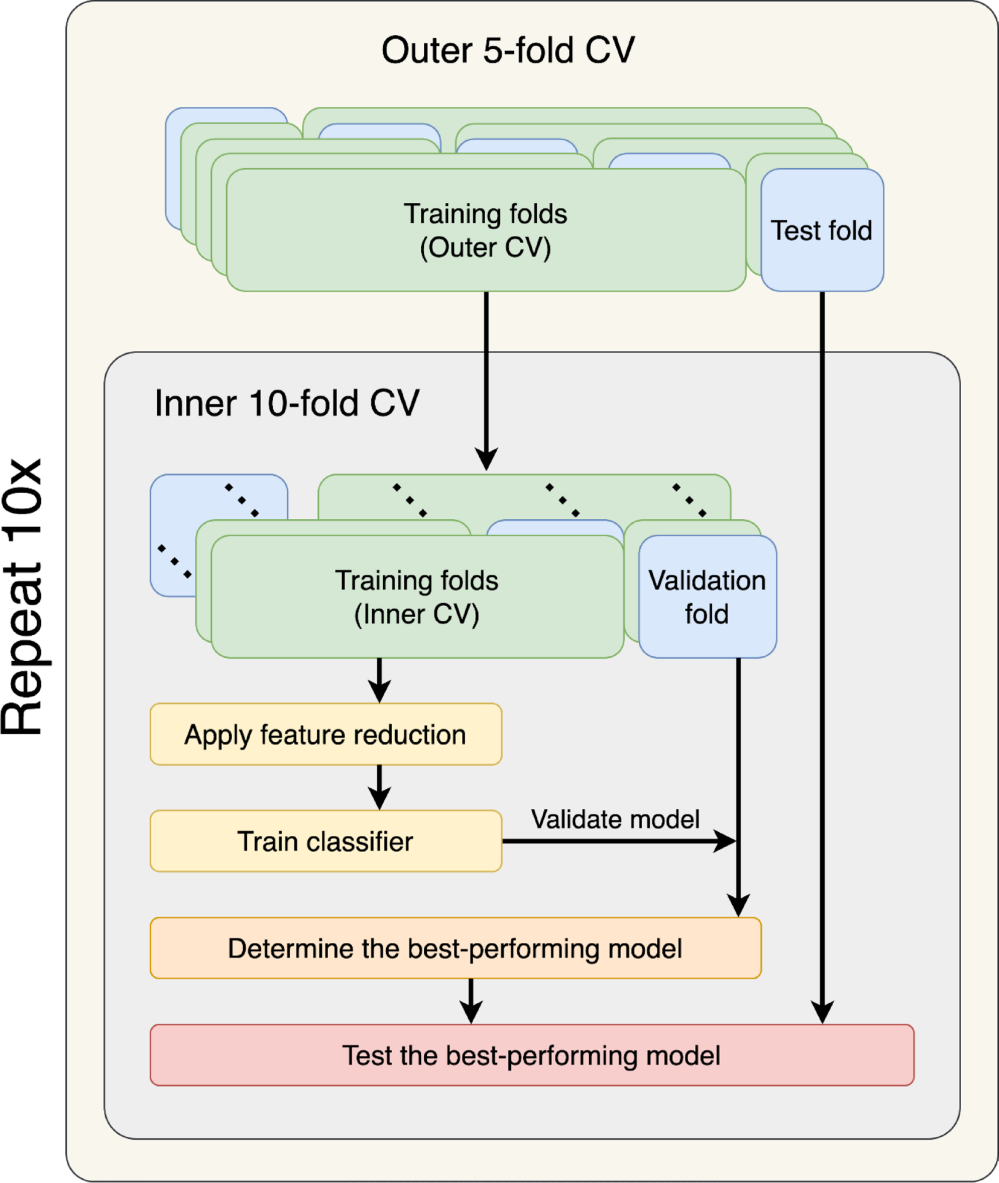
Overall study design. Nested cross-validation was applied to each data set. In the first step, the data were split into five folds, four of which were used for training, and one was retained for testing. On the four training folds, another 10-fold cross-validation was applied, i.e., the data were split into ten folds, nine of which were used for training and one for validation. In the inner cross-validation, the best hyperparameters are determined. The best-performing model was then retrained on the training data and evaluated on the retained test fold. The resulting predictions were pooled to obtain a single AUC. This process was then repeated ten times.

This study systematically evaluated the performance of feature projection versus feature selection methods in radiomic classification tasks. A comprehensive collection of 50 publicly available radiomic datasets were used, each consisting exclusively of radiomic features. To assess the impact of feature projection techniques, a rigorous nested cross-validation strategy was implemented (Fig. 6), which includes a stratified outer 5-fold CV for performance estimation and an inner 10-fold CV for hyperparameter tuning and model selection. Models using four common machine learning classifiers, training a model for each combination of feature reduction method and classifier, were created. Hyperparameters were optimized using grid search within the inner CV loop. Model performance was evaluated using multiple metrics, with emphasis on the AUC. Statistical comparisons were performed using the Friedman test with the post-hoc Nemenyi test. Differences between both approaches were also compared using a Bland-Altman type analysis. In addition, it was examined how dataset characteristics (sample size, number of features, dimensionality, and class balance) influenced differences between the two techniques. Finally, the computational efficiency was assessed by considering the trade-offs between performance and computational time, and by measuring runtimes across varying feature set sizes.

### Datasets

The radMLBench collection^41^which is a collection of 50 publicly available radiomic datasets in tabular form (Table 1). The datasets contain only radiomic features; other features, such as clinical parameters or genetic factors, were removed. All datasets were normalized using z-Scores, i.e., each feature has mean of 0 and variance of 1. More details about each dataset can be found in the radMLBench repository and in the corresponding studies.

**Table 1:** Overview of all datasets employed.

**Dataset**	**Modality**	**Outcome**	**Number of Instances**	**Positive Instances**	**Negative Instances**	**Number of features**	**Dimensionality**	**Balance [%]**
Ahn2021	MRI	MGMT mutation status	114	63	51	108	0.96	55
Arita2018	MRI	IDH mutation status	168	111	57	683	4.08	66
BraTS-2021	MRI	MGMT mutation status	577	301	276	4060	7.04	52
Brancato2023	MRI	Clinical significance	58	35	23	2380	41.07	60
C4KC-KiTS	CT	Histological subtype	70	46	24	315	4.53	66
Colorectal-Liver-Metastases	CT	Survival (at 10 years)	90	76	14	525	5.86	84
Dai2023	CT	Thrombocytopenia presence	119	31	88	851	7.17	26
Deng2023	MRI	Pathological diagnosis	261	94	167	225	0.87	36
Dong2022	CT	Tuberculosis granuloma presence	279	136	143	851	3.06	49
Fusco2022	MRI	Malignancy presence	54	33	21	192	3.59	61
Granata2021	CT	Overall survival (at 32 months)	88	41	47	580	6.61	47
Granata2024	MRI	Tumor budding presence	51	38	13	851	16.73	75
HCC-TACE-Seg	CT	Lymph node metastasis presence	84	12	72	420	5.02	14
HNSCC	CT	Lymph node metastasis presence	93	25	68	105	1.15	27
Head-Neck-PET-CT	PET/CT	Lymph node metastasis presence	91	61	30	210	2.33	67
Head-Neck-Radiomics-HN1	CT	Lymph node metastasis presence	137	61	76	105	0.78	45
Hosny2018A	CT	Overall survival (at 2 years)	293	159	134	984	3.37	54
Hosny2018B	CT	Overall survival (at 2 years)	207	60	147	984	4.76	29
Hosny2018C	CT	Overall survival (at 2 years)	183	133	50	984	5.39	73
Huang2023	CT	Malignancy presence	212	97	115	855	4.04	46
Hunter2023	CT	Nodule malignancy presence	520	282	238	1998	3.85	54
ISPY1	MRI	Hormone receptor positive status	161	92	69	370	2.31	57
Keek2020	CT	Overall survival (at 3 years)	273	119	154	1322	4.85	44
LGG-1p19qDeletion	MRI	1p19q co-deletion status	159	102	57	2030	12.78	64
LNDb	CT	Fleischner score group	173	115	58	105	0.62	66
Li2020	MRI	Immunohistochemical result	51	32	19	396	7.8	63
Lu2019	CT	Progression free survival (at 2 years)	75	55	20	657	8.79	73
Meningioma-SEG-CLASS	MRI	Tumor grade	88	38	50	2030	23.09	43
NSCLC-Radiogenomics	PET/CT	EGFR mutation status	144	23	121	105	0.74	16
OcanaTienda2023	MRI	Survival (at 2 years)	67	32	35	1130	16.9	48
PI-CAI	MRI	Gleason score risk group	969	637	332	3045	3.14	66
Petrillo2023	MRI	Luminal type presence	128	47	81	851	6.66	37
Prostate-MRI-US-Biopsy	MRI	Gleason score risk group	773	596	177	1015	1.32	77
QIN-HEADNECK	PET/CT	Lymph node metastasis presence	59	44	15	210	3.59	75
Ramella2018	CT	Adaptive chemoradiation presence	91	50	41	242	2.68	55
Sasaki2019	MRI	MGMT mutation status	138	68	70	587	4.27	49
Song2020	MRI	Clinical significance	260	127	133	264	1.02	49
UCSF-PDGM	MRI	IDH mutation status	418	373	45	7105	17	89
UPENN-GBM	MRI	MGMT mutation status	187	79	108	11165	59.72	42
Veeraraghavan2020	MRI	High tumor burden presence	150	47	103	200	1.35	31
WORC-CRLM	CT	Histopathological growth pattern	77	37	40	1015	13.21	48
WORC-Desmoid	MRI	Fibromatosis presence	203	72	131	1015	5.01	35
WORC-GIST	CT	Gastrointestinal stromal tumors presence	245	124	121	1015	4.15	51
WORC-Lipo	MRI	Malignancy presence	114	57	57	1015	8.92	50
WORC-Liver	MRI	Malignancy presence	186	94	92	1015	5.47	51
WORC-Melanoma	CT	BRAF mutation status	95	47	48	1015	10.71	49
Wang2024	MRI	EGFR mutation status	67	27	40	280	4.21	40
Zhang2023	CT	Histological invasiveness	203	104	99	1781	8.78	51
Zhang2024A	PET/CT	Histological type	255	145	110	3850	15.11	57
Zhang2024B	CT	Lymph node metastasis presence	192	126	66	833	4.35	66

Dimensionality denotes the number of features divided by the number of instances. Balance denotes the percentage of positive instances in the dataset.

### Feature reduction

Then, either a feature selection or projection method was applied to the data. Nine feature projection methods were utilized^42^: Principal Component Analysis (PCA), Kernel PCA (kPCA), Independent Component Analysis (ICA), Factor Analysis (FA), Non-Negative Matrix Factorization (NMF), Mini-Batch Dictionary Learning (MBDL), Truncated Singular Value Decomposition (SVD), Uniform Manifold Approximation and Projection (UMAP), and Supervised Random Projection (SRP) (see the Supplementary Materials for a more detailed description). These methods were mainly chosen because they are widely used, well-tested, and have fast, reliable implementations available in the scikit-learn library which was employed for all experiments.

For comparison, following feature selection methods were tested^43,44^: Least Absolute Shrinkage and Selection Operator (LASSO), Minimum Redundancy Maximum Relevance ensemble (MRMRe), Bhattacharyya distance, Kendall’s rank correlation, t-Score, Recursive Feature Elimination based on Logistic Regression (RFE-LR), Boruta, and Extremely Randomized Trees (ET) (Supplementary Materials). These methods were chosen because they are either widely used in radiomics studies or have shown good performance^16^.

However, the application of these methods is not straightforward since they have several hyperparameters, some of which influence their performance strongly. For example, kPCA operates on a kernel function that must be selected in advance and may have parameters that determine its shape. Similarly, LASSO has a hyperparameter, lambda, that controls the strength of the regularization.

Since hyperparameter selection can be computationally expensive, their default values, which are typically chosen for optimal performance across a large number of datasets, were not changed. This approach also reflects current practice in radiomics research. However, to avoid computational overhead, it was necessary to fix a lower step size parameter for RFE, which controls the number of features retained at each step. This parameter was set to 10%.

Additionally, none of the reduction methods used in this study inherently provide an optimal number of features to retain. For example, PCA can be configured to either select a fixed number of features or to retain a certain amount of variance. LASSO can determine an optimal number of features when a sparsity parameter is provided. Therefore, in the experiments, the number of retained features was fixed for all methods and values between *N* = 1, 2, 4, 8, 16, and 32 were tested. The values were chosen on a logarithmic scale because this allows exploration of the parameter space while maintaining computational efficiency.

### Classifiers

From the retained features, classifiers were trained using common machine learning methods: Naive Bayes (NB), which uses Bayes’ theorem to compute the probability of the class given the features; Logistic Regression (LR), which models the probability of a class via a linear model and a logistic function as the link function; Support Vector Machines (SVM) with a Radial-Basis-Function (RBF) kernel, which projects the data into a high-dimensional space and trains a linear classifier based on the maximum margin principle. and Random Forest (RF), which trains an ensemble of decision trees. Except for NB, each method has several hyperparameters, and only one was optimized for this study: the regularization parameter C for the logistic regression and the SVM, both chosen among 2^−7^, 2^−5^, … 2^5^, 2^7^; the number of trees for the RF, chosen among 10, 25, 50, 100, 250, 500, and 1000. More details can be found in the code.

### Training

During training, models were created for each combination of feature reduction methods and classifier models. To obtain reliable results, a nested cross-validation (CV) strategy was used (Fig. 6). First, the dataset was divided into five outer folds, with each fold being used once as a test set. On the remaining four folds, which formed the training data, a model was trained using an ‘inner’ 10-fold CV. Thus, the training data was split into ten inner folds, with each fold used once for validation and the other nine used for training.

The splitting was done in a stratified manner, i.e., the balance between positive and negative classes is approximately the same in each fold. This is necessary because metrics like the AUC cannot be computed if a fold happens to have samples from only one class. On the training set, all hyperparameters were then tested using a simple grid search: For each feature reduction method (i.e., either selection or projection), combinations of number of features, classifiers, and their hyperparameters were tested. In addition, the performance of not reducing the features was also measured. The predictions were then pooled across the ten validation folds to obtain an inner AUC. The best performing model was then selected and retrained on all training data of the outer CV, i.e., all data except the outer test fold. The retrained model was then evaluated on the outer test fold. These predictions on the outer folds were then pooled to obtain a single metric for each feature reduction method. The whole process was then repeated ten times for each dataset.

### Evaluation

After training, the overall mean performance of each feature reduction method was computed by averaging the metrics across all datasets. Five evaluation metrics were used: AUC, AUPRC, and F1, F0.5, and F2 scores. While AUC is the de facto standard in radiomics, AUPRC and F scores are particularly useful for imbalanced datasets, as they better account for the trade-off between precision and recall.

To assess the relative performance, methods were ranked by computing their relative rank on each dataset and then averaging these ranks across all datasets. Additionally, the improvement in performance compared to using no feature reduction was calculated for each method. To statistically compare the methods, a Friedman test was conducted to detect significant differences in performance^45^. If a significant difference was observed, a post-hoc Nemenyi test was computed to identify pairwise differences.

To directly compare feature projection with feature selection methods, the best-performing methods were determined for each dataset. Then, a Bland-Altman-type analysis was conducted to assess the magnitude of differences. For each dataset, the average and the difference were calculated and plotted, and the mean difference along with the limits of agreement (LoA), defined as the mean difference ± 1.96 standard deviations, was reported. The differences were also tested using a Wilcoxon signed-rank test to determine whether the two reduction methods were statistically different.

To determine the relative gain that would be achieved by replacing one method with another, a table was created showing both the average gain and the number of datasets on which the other method performed better.

In addition, linear regression was used to understand whether the observed differences in the metrics might depend on the number of features, the number of instances, the dimensionality of the datasets, or the class balance, indicating in which scenario feature projection or feature selection might be more useful.

Finally, the computational efficiency of each method was evaluated by plotting the average performance across all datasets against computational time, with a Pareto front highlighting the optimal trade-offs between accuracy and speed. Additionally, the average runtimes across each dataset and each feature reduction method for each *N* = 1, 2, …, 32 were visualized.

### Statistical analysis

P values below 0.05 were considered to be significant. Due to the exploratory nature of this study, no corrections were made for multiple testing. All analyses were performed using Python 3.10 and the scipy, scikit-learn, and pyMRMRe modules. The implementation of SRP by Ghojogh and Crawley was used^18^. All data and code will be made publicly available on github.

## Supplementary Information

Below is the link to the electronic supplementary material.

Supplementary Material 1

## Data Availability

The datasets generated and/or analyzed during the current study are available in the github repository at https://github.com/aydindemircioglu/radPCA.
