# Supplementary material for: Benchmarking feature projection methods in radiomics
Source: Sci Rep. 2025 Sep 5;15:32368. doi: 10.1038/s41598-025-16070-w (PMC12413460; doi:10.1038/s41598-025-16070-w)
Supplement: Supplementary file 1 — Supplementary Material 1 [file 41598_2025_16070_MOESM1_ESM.pdf]

Benchmarking feature projection methods in radiomics

Aydin Demircioğlu, PhD[1, *]

Supplementary Materials

Feature projection methods

Principal Component Analysis

PCA is a linear technique that seeks to identify the principal components, which are orthogonal directions in the feature space that maximize the variance of the data[1]. By projecting the data onto a lower-dimensional subspace spanned by the top principal components, PCA can be used as a feature reduction method.

Kernel PCA

Kernel PCA extends PCA by first implicitly mapping the data into a higher-dimensional feature space using a kernel function[2]. PCA is then performed in this kernel space. This allows kPCA to capture non-linear relationships in the data that might be missed by linear PCA. The kernel trick[3] makes the computation in the high-dimensional spaces feasible.

Independent Component Analysis

ICA is a technique that aims to separate a multivariate signal into additive subcomponents that are statistically independent[4]. Unlike PCA, which focuses on maximizing variance, ICA seeks

components that are non-Gaussian. Similar to PCA, it can be used as a feature reduction method by considering only the top components.

Factor Analysis

FA is a statistical method that assumes the observed variables can be explained by a smaller number of latent, unobserved factors[5]. It models the relationships between observed variables and these underlying factors. By using the latent factors as features, FA can be utilized as a feature reduction method.

Non-Negative Matrix Factorization

NMF is a decomposition technique that factorizes a non-negative matrix into two lower-rank non-negative matrices[6,7]. The non-negativity constraint allows for a parts-based representation of the data, where the original features are represented as additive combinations of the components learned by NMF. It can be applied in radiomics by normalizing the features using the Min-Max method.

Mini-Batch Dictionary Learning

Dictionary Learning (DL) is a method that learns a set of representative vectors, called a dictionary, such that input data can be approximated as sparse linear combinations of these vectors[8]. Since this process can be computationally intensive, MBDL is a variant that updates the dictionary using small subsets of the data at each step. The learned dictionary can then serve as a new feature space for dimensionality reduction.

Truncated Singular Value Decomposition

In Truncated SVD, the data matrix is approximated with a lower-rank matrix by keeping only the largest singular values and their corresponding singular vectors[9]. It can be used as feature reduction method by retaining the components associated with the largest singular values.

Uniform Manifold Approximation and Projection

UMAP is primarily a visualization technique[10], however, it can also be used for dimensionality reduction by constructing a high-dimensional graph of the data and optimizing a low-dimensional embedding that preserves the topological structure.

Supervised Random Projection

SRP can be understood as a computationally inexpensive variant of supervised PCA (sPCA), which extends PCA by incorporating the target, leading to components that not only capture the most variance but are also most predictive[11,12]. SRP differs from sPCA by projecting the data onto a randomly chosen subspace that is weakly supervised, which leads to computation efficiency.

Feature selection methods

Least Absolute Shrinkage and Selection Operator

LASSO is a linear regression-based method that adds an L1 penalty to the loss function[13], which encourages sparsity in the model coefficients, leading to exclusion of non-informative features and reduction of overfitting.

Minimum Redundancy Maximum Relevance ensemble

Minimum Redundancy Maximum Relevance (MRMR) is a method that employs mutual information to select features that are highly relevant to the target variable while minimizing redundancy[14]. Since MRMR often yields instable results, it can be used in an ensemble approach, increasing stability and robustness of the selected feature set[15].

Bhattacharyya distance

The Bhattacharyya distance aims to measure the similarity between two probability distributions[16]. The distance was adapted for feature selection[17], where features are ranked based on the distance between the class distributions.

Kendall's rank correlation

Kendall's tau[18,19] measures the correlation between two rankings and can be used for feature selection by computing the correlation of each feature with the target and using it as a surrogate for feature importance.

t-Score

The t-Score method applies an independent t-test to each feature, comparing its values across different outcome classes. By computing the significance of each feature with the target, the t-Score can be used for feature selection.

Recursive Feature Elimination based on Logistic Regression

Recursive Feature Elimination (RFE) is a wrapper method that first employs a classifier to obtain weights for each feature[20]. The features with the lowest absolute coefficients are then removed. The method is then applied recursively until a specified number of features are retained. RFE-LR uses logistic regression (LR) as a classifier.

Boruta

Boruta is a feature selection algorithm that assesses the importance of actual features with that of randomly permuted features[21], where the importance is measured using random forests. The procedure is repeated and only those features are retained that consistently outperform the randomized features.

Extremely Randomized Trees

Extremely Randomized Trees (ET) is an ensemble method that constructs multiple decision trees using random split points, increasing the diversity of the trees[22]. Feature importance is then determined by how often each tree is used for splitting across the trees.

Ranking of the methods

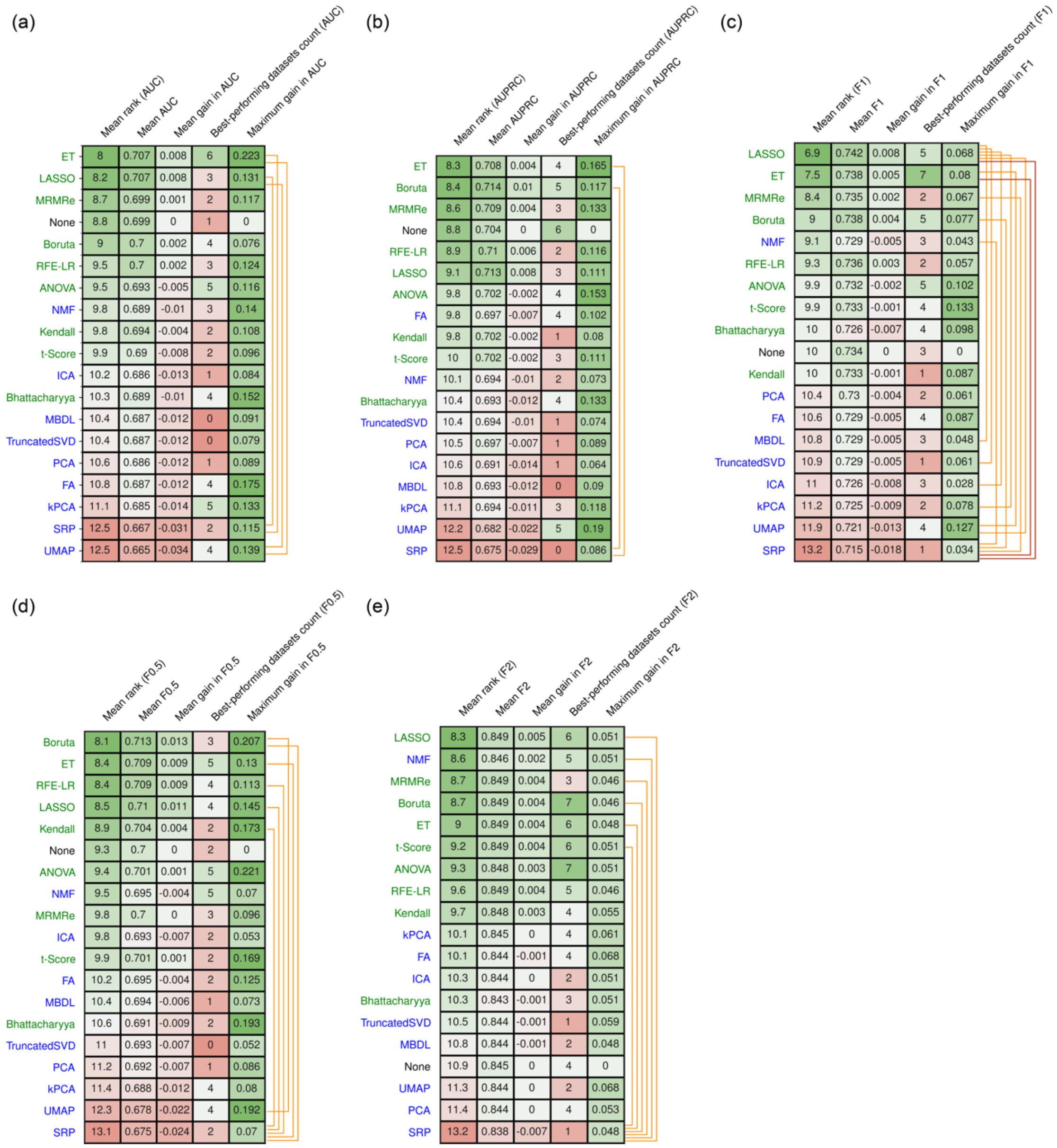

Figure S1 Ranking of feature reduction methods.

Ranking of feature reduction methods with respect to (a) AUC, (b) AUPRC, (c) F1 score, (d) F0.5 score, and (e) F2 score. Methods in green represent feature selection, while those in blue represent feature projection methods. The mean rank was calculated by ranking all methods for each dataset and averaging the ranks. The mean gain was calculated as the difference in performance compared to no feature reduction (None). The number of best performing datasets indicates the number of datasets on which the corresponding method performed best. The maximum gain represents the highest performance improvement observed across all datasets compared to using no feature reduction. The methods were tested for differences using a Friedman test followed by a post-hoc Nemenyi test. Methods with significant differences are connected by an orange line ($p < 0.05$) or a red line ($p < 0.001$).

Bland-Altmann plots

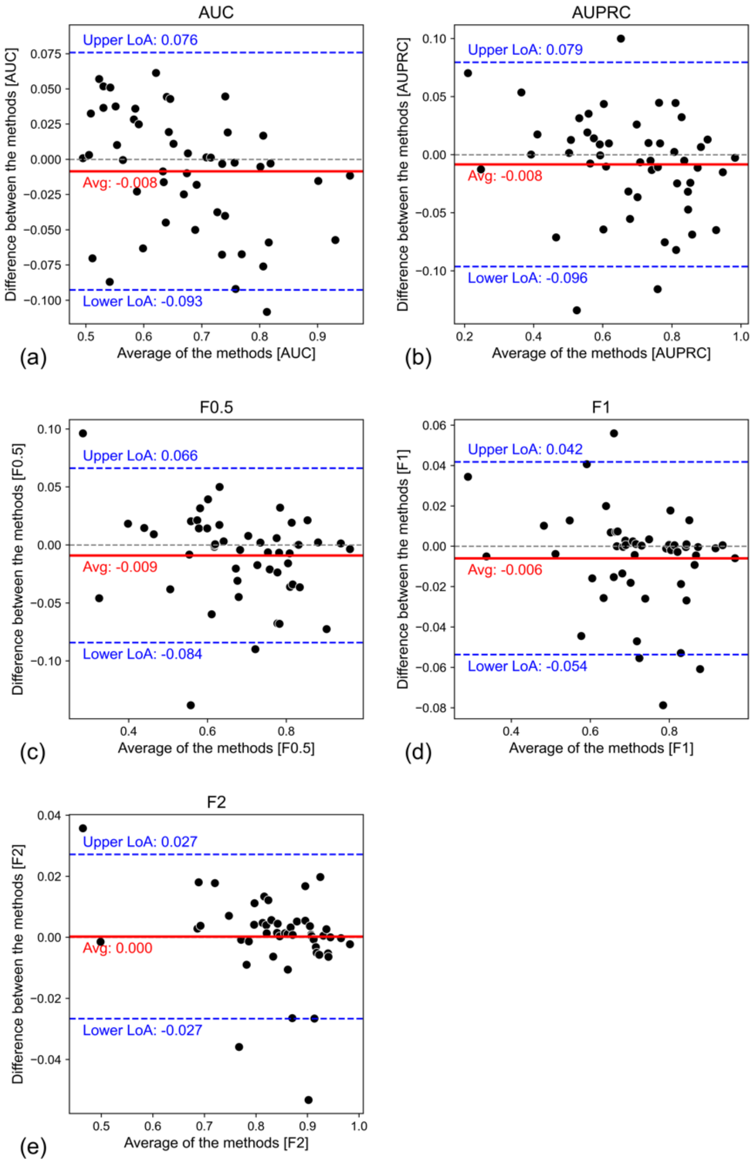

Figure S2 Bland-Altman plots comparing projection and selection methods.

Bland-Altman plots showing the agreement between projection and selection methods for (a) AUC, (b) AUPRC, (c) F1 score, (d) F0.5 score, and (e) F2 score on each dataset. Each plot displays the difference between the two methods against their average. The solid red line indicates the mean difference, while the blue dashed lines represent the upper and lower limits of agreement (LoA), which are calculated as the mean difference ±1.96 times the standard deviation of the differences. A positive average difference indicates that at least one feature projection method outperformed all selection methods for that dataset, whereas a negative difference indicates that at least one feature selection method outperformed all projection methods.

Benefit and gain comparisons in AUPRC

	SRP	UMAP	kPCA	MBDL	ICA	PCA	TruncatedSVD	Bhattacharyya	NMF	t-Score	Kendall	FA	ANOVA	LASSO	RFE-LR	None	MRMRe	Boruta	ET
ET	-16 (-0.033)	-20 (-0.026)	-14 (-0.014)	-16 (-0.015)	-12 (-0.017)	-12 (-0.011)	-14 (-0.014)	-8 (-0.015)	-10 (-0.014)	-9 (-0.006)	-4 (-0.006)	-4 (-0.011)	-9 (-0.006)	-12 (0.005)	-4 (0.002)	-8 (-0.004)	-2 (0.001)	2 (0.006)	
Boruta	-16 (-0.039)	-14 (-0.032)	-8 (-0.02)	-14 (-0.022)	-14 (-0.023)	-20 (-0.017)	-8 (-0.02)	-6 (-0.021)	-6 (-0.02)	-10 (-0.012)	-6 (-0.012)	-6 (-0.017)	-14 (-0.012)	-4 (-0.001)	-2 (-0.004)	-6 (-0.01)	-6 (-0.005)		-2 (-0.006)
MRMRe	-18 (-0.034)	-12 (-0.026)	-10 (-0.015)	-14 (-0.016)	-16 (-0.018)	-14 (-0.012)	-10 (-0.014)	-12 (-0.016)	-6 (-0.015)	-10 (-0.007)	-6 (-0.006)	-4 (-0.012)	-8 (-0.007)	-4 (0.004)	-2 (0.002)	-2 (-0.004)		6 (0.005)	2 (-0.001)
None	-18 (-0.029)	-16 (-0.022)	-16 (-0.011)	-12 (-0.012)	-12 (-0.014)	-8 (-0.007)	-6 (-0.01)	-8 (-0.012)	-8 (-0.01)	-6 (-0.002)	-8 (-0.002)	-2 (-0.007)	-10 (-0.002)	0 (0.008)	-2 (0.006)		2 (0.004)	6 (0.01)	8 (0.004)
RFE-LR	-18 (-0.035)	-16 (-0.028)	-12 (-0.016)	-12 (-0.018)	-8 (-0.019)	-8 (-0.013)	-8 (-0.016)	-14 (-0.017)	-10 (-0.016)	-10 (-0.008)	-2 (-0.008)	-4 (-0.013)	-2 (-0.008)	0 (0.003)		2 (-0.006)	2 (-0.002)	2 (0.004)	4 (-0.002)
LASSO	-16 (-0.038)	-16 (-0.03)	-14 (-0.019)	-12 (-0.02)	-10 (-0.022)	-6 (-0.016)	-6 (-0.018)	-12 (-0.02)	-6 (-0.019)	-2 (-0.011)	0 (-0.01)	-8 (-0.016)	0 (-0.011)		0 (-0.003)	0 (-0.008)	4 (-0.004)	4 (0.001)	12 (-0.005)
ANOVA	-14 (-0.027)	-14 (-0.02)	-10 (-0.008)	-2 (-0.009)	-8 (-0.011)	-2 (-0.005)	-2 (-0.008)	-4 (-0.009)	0 (-0.008)	-1 (0.0)	-2 (0.0)	0 (-0.005)		0 (0.011)	2 (0.008)	10 (0.002)	8 (0.007)	14 (0.012)	9 (0.006)
FA	-6 (-0.022)	-6 (-0.015)	0 (-0.003)	-4 (-0.004)	-8 (-0.006)	-6 (-0.0)	-4 (-0.003)	-6 (-0.004)	0 (-0.003)	-6 (0.005)	-2 (0.005)		0 (0.005)	8 (0.016)	4 (0.013)	2 (0.007)	4 (0.012)	6 (0.017)	4 (0.011)
Kendall	-16 (-0.027)	-14 (-0.02)	-2 (-0.008)	-2 (-0.01)	0 (-0.011)	-6 (-0.005)	-6 (-0.008)	2 (-0.01)	-8 (-0.008)	2 (-0.0)		2 (-0.005)	2 (-0.0)	0 (0.01)	2 (0.008)	8 (0.002)	6 (0.006)	6 (0.012)	4 (0.006)
t-Score	-12 (-0.027)	-6 (-0.02)	-8 (-0.008)	-4 (-0.009)	-2 (-0.011)	-10 (-0.005)	-10 (-0.008)	4 (-0.009)	0 (-0.008)		-2 (0.0)	6 (-0.005)	1 (-0.0)	2 (0.011)	10 (0.008)	6 (0.002)	10 (0.007)	10 (0.012)	9 (0.006)
NMF	-24 (-0.019)	-8 (-0.012)	-6 (-0.0)	0 (-0.001)	-8 (-0.003)	4 (0.003)	2 (0.0)	-6 (-0.001)		0 (0.008)	8 (0.008)	0 (0.003)	0 (0.008)	6 (0.019)	10 (0.016)	8 (0.01)	6 (0.015)	6 (0.02)	10 (0.014)
Bhattacharyya	-16 (-0.018)	-14 (-0.01)	6 (0.001)	-4 (-0.0)	0 (-0.002)	-4 (0.004)	-2 (0.002)		6 (0.001)	-4 (0.009)	-2 (0.01)	6 (0.004)	4 (0.009)	12 (0.02)	14 (0.017)	8 (0.012)	12 (0.016)	6 (0.021)	8 (0.015)
TruncatedSVD	-8 (-0.019)	-16 (-0.012)	-6 (-0.001)	-6 (-0.002)	-2 (-0.004)	0 (0.003)		2 (-0.002)	-2 (-0.0)	10 (0.008)	6 (0.008)	4 (0.003)	2 (0.008)	6 (0.018)	8 (0.016)	6 (0.01)	10 (0.014)	8 (0.02)	14 (0.014)
PCA	-16 (-0.022)	-8 (-0.015)	-6 (-0.003)	-12 (-0.004)	-4 (-0.006)		0 (-0.003)	4 (-0.004)	-4 (-0.003)	10 (0.005)	6 (0.005)	6 (0.0)	2 (0.005)	6 (0.016)	8 (0.013)	8 (0.007)	14 (0.012)	20 (0.017)	12 (0.011)
ICA	-12 (-0.016)	-10 (-0.008)	-8 (0.003)	-10 (0.002)		4 (0.006)	2 (0.004)	0 (0.002)	8 (0.003)	2 (0.011)	0 (0.011)	8 (0.006)	8 (0.011)	10 (0.022)	8 (0.019)	12 (0.014)	16 (0.018)	14 (0.023)	12 (0.017)
MBDL	-22 (-0.018)	-12 (-0.01)	-14 (0.001)		10 (-0.002)	12 (0.004)	6 (0.002)	4 (0.0)	0 (0.001)	4 (0.009)	2 (0.01)	4 (0.004)	2 (0.009)	12 (0.02)	12 (0.018)	12 (0.012)	14 (0.016)	14 (0.022)	16 (0.015)
kPCA	-8 (-0.019)	-12 (-0.011)		14 (-0.001)	8 (-0.003)	6 (0.003)	6 (0.001)	-6 (-0.001)	6 (0.0)	8 (0.008)	2 (0.008)	0 (0.003)	10 (0.008)	14 (0.019)	12 (0.016)	16 (0.011)	10 (0.015)	8 (0.02)	14 (0.014)
UMAP	2 (-0.007)		12 (0.011)	12 (0.01)	10 (0.008)	8 (0.015)	16 (0.012)	14 (0.01)	8 (0.012)	6 (0.02)	14 (0.02)	6 (0.015)	14 (0.02)	16 (0.03)	16 (0.028)	16 (0.022)	12 (0.026)	14 (0.032)	20 (0.026)
SRP		-2 (0.007)	8 (0.019)	22 (0.018)	12 (0.016)	16 (0.022)	8 (0.019)	16 (0.018)	24 (0.019)	12 (0.027)	16 (0.027)	6 (0.022)	14 (0.027)	16 (0.038)	18 (0.035)	18 (0.029)	18 (0.034)	16 (0.039)	16 (0.033)

Figure S3A Benefit and gain comparison in AUPRC

The top number in each cell indicates the number of datasets, where replacing one method with another method results in a gain or loss in performance. The number in parentheses represents the average gain in this case.

Benefit and gain comparisons in F1 score

	SRP	UMAP	kPCA	ICA	TruncatedSVD	MBDL	FA	PCA	Kendall	None	Bhattacharyya	t-Score	ANOVA	RFE-LR	NMF	Boruta	MRMRe	ET	LASSO
LASSO	-33 (-0.027)	-20 (-0.021)	-20 (-0.017)	-19 (-0.016)	-23 (-0.013)	-21 (-0.013)	-20 (-0.013)	-19 (-0.012)	-18 (-0.009)	-16 (-0.008)	-16 (-0.016)	-19 (-0.009)	-13 (-0.01)	-18 (-0.006)	-10 (-0.013)	-13 (-0.004)	-13 (-0.007)	-1 (-0.004)	
ET	-26 (-0.023)	-21 (-0.018)	-20 (-0.013)	-17 (-0.013)	-16 (-0.01)	-18 (-0.009)	-22 (-0.009)	-18 (-0.008)	-8 (-0.006)	-17 (-0.005)	-12 (-0.012)	-9 (-0.005)	-9 (-0.006)	-12 (-0.002)	-11 (-0.009)	-7 (-0.001)	-9 (-0.003)		1 (0.004)
MRMRe	-29 (-0.02)	-16 (-0.015)	-11 (-0.01)	-14 (-0.01)	-15 (-0.007)	-17 (-0.006)	-19 (-0.006)	-11 (-0.005)	-5 (-0.003)	-6 (-0.002)	-7 (-0.009)	-12 (-0.002)	-17 (-0.003)	-7 (0.001)	-3 (-0.006)	4 (0.002)		9 (0.003)	13 (0.007)
Boruta	-15 (-0.022)	-11 (-0.017)	-11 (-0.012)	-13 (-0.012)	-8 (-0.009)	-9 (-0.008)	-10 (-0.009)	-11 (-0.007)	-11 (-0.005)	-11 (-0.004)	-5 (-0.011)	2 (-0.004)	4 (-0.005)	-1 (-0.001)	-1 (-0.008)		-4 (-0.002)	7 (0.001)	13 (0.004)
NMF	-18 (-0.014)	-14 (-0.009)	-19 (-0.004)	-10 (-0.004)	-10 (-0.001)	-7 (-0.0)	-14 (-0.0)	-6 (0.001)	-7 (0.004)	-2 (0.005)	-10 (-0.003)	0 (0.004)	1 (0.003)	4 (0.007)		1 (0.008)	3 (0.006)	11 (0.009)	10 (0.013)
RFE-LR	-27 (-0.021)	-14 (-0.016)	-7 (-0.011)	-3 (-0.011)	-6 (-0.008)	-8 (-0.007)	-5 (-0.007)	0 (-0.006)	-10 (-0.004)	-5 (-0.003)	-4 (-0.01)	-4 (-0.003)	-7 (-0.004)		-4 (-0.007)	1 (0.001)	7 (-0.001)	12 (0.002)	18 (0.006)
ANOVA	-15 (-0.017)	-14 (-0.012)	-8 (-0.007)	-3 (-0.007)	-5 (-0.004)	-1 (-0.003)	0 (-0.003)	-6 (-0.002)	-3 (0.001)	-4 (0.002)	4 (-0.006)	4 (0.001)		7 (0.004)	-1 (-0.003)	-4 (0.005)	17 (0.003)	9 (0.006)	13 (0.01)
t-Score	-18 (-0.018)	-7 (-0.013)	-4 (-0.008)	-6 (-0.007)	-8 (-0.004)	-7 (-0.004)	0 (-0.004)	-4 (-0.003)	1 (-0.0)	1 (0.001)	5 (-0.007)		-4 (-0.001)	4 (0.003)	0 (-0.004)	-2 (0.004)	12 (0.002)	9 (0.005)	19 (0.009)
Bhattacharyya	-15 (-0.011)	-14 (-0.006)	-7 (-0.001)	-4 (-0.001)	-1 (0.002)	-2 (0.003)	-5 (0.003)	-2 (0.004)	2 (0.006)	0 (0.007)		-5 (0.007)	-4 (0.006)	4 (0.01)	10 (0.003)	5 (0.011)	7 (0.009)	12 (0.012)	16 (0.016)
None	-9 (-0.018)	-10 (-0.013)	-10 (-0.009)	-3 (-0.008)	-7 (-0.005)	-12 (-0.005)	-5 (-0.005)	-2 (-0.004)	2 (-0.001)		0 (-0.007)	-1 (-0.001)	4 (-0.002)	5 (0.003)	2 (-0.005)	11 (0.004)	6 (0.002)	17 (0.005)	16 (0.008)
Kendall	-19 (-0.017)	-13 (-0.012)	-5 (-0.008)	-4 (-0.007)	-2 (-0.004)	-9 (-0.004)	-9 (-0.004)	2 (-0.003)		-2 (0.001)	-2 (-0.006)	-1 (0.0)	3 (-0.001)	10 (0.004)	7 (-0.004)	11 (0.005)	5 (0.003)	8 (0.006)	18 (0.009)
PCA	-14 (-0.015)	-15 (-0.01)	-7 (-0.005)	0 (-0.004)	-8 (-0.001)	-2 (-0.001)	5 (-0.001)		-2 (0.003)	2 (0.004)	2 (-0.004)	4 (0.003)	6 (0.002)	0 (0.006)	6 (-0.001)	11 (0.007)	11 (0.005)	18 (0.008)	19 (0.012)
FA	-14 (-0.014)	-6 (-0.008)	-6 (-0.004)	-6 (-0.003)	-7 (-0.0)	-8 (0.0)		-5 (0.001)	9 (0.004)	5 (0.005)	5 (-0.003)	0 (0.004)	0 (0.003)	5 (0.007)	14 (0.0)	10 (0.009)	19 (0.006)	22 (0.009)	20 (0.013)
MBDL	-15 (-0.014)	-5 (-0.009)	-8 (-0.004)	-6 (-0.003)	-2 (-0.001)		8 (-0.0)	2 (0.001)	9 (0.004)	12 (0.005)	2 (-0.003)	7 (0.004)	1 (0.003)	8 (0.007)	7 (0.0)	9 (0.008)	17 (0.006)	18 (0.009)	21 (0.013)
TruncatedSVD	-18 (-0.013)	-5 (-0.008)	-2 (-0.004)	-3 (-0.003)		2 (0.001)	7 (0.0)	8 (0.001)	2 (0.004)	7 (0.005)	1 (-0.002)	8 (0.004)	5 (0.004)	6 (0.008)	10 (0.001)	8 (0.009)	15 (0.007)	16 (0.01)	23 (0.013)
ICA	-5 (-0.01)	-7 (-0.005)	-1 (-0.001)		3 (0.003)	6 (0.003)	6 (0.003)	0 (0.004)	4 (0.007)	3 (0.008)	4 (0.001)	6 (0.007)	3 (0.007)	3 (0.011)	10 (0.004)	13 (0.012)	14 (0.01)	17 (0.013)	19 (0.016)
kPCA	-15 (-0.01)	-11 (-0.005)		1 (0.001)	2 (0.004)	8 (0.004)	6 (0.004)	7 (0.005)	5 (0.008)	10 (0.009)	7 (0.001)	4 (0.008)	8 (0.007)	7 (0.011)	19 (0.004)	11 (0.012)	11 (0.01)	20 (0.013)	20 (0.017)
UMAP	-15 (-0.005)		11 (0.005)	7 (0.005)	5 (0.008)	5 (0.009)	6 (0.008)	15 (0.01)	13 (0.012)	10 (0.013)	14 (0.006)	7 (0.013)	14 (0.012)	14 (0.016)	14 (0.009)	11 (0.017)	16 (0.015)	21 (0.018)	20 (0.021)
SRP		15 (0.005)	15 (0.01)	5 (0.01)	18 (0.013)	15 (0.014)	14 (0.014)	14 (0.015)	19 (0.017)	9 (0.018)	15 (0.011)	18 (0.018)	15 (0.017)	27 (0.021)	18 (0.014)	15 (0.022)	29 (0.02)	26 (0.023)	33 (0.027)

Figure S3B Benefit and gain comparison in F1 score

The top number in each cell indicates the number of datasets, where replacing one method with another method results in a gain or loss in performance. The number in parentheses represents the average gain in this case.

Benefit and gain comparisons in F0.5 score

	SRP	UMAP	kPCA	PCA	TruncatedSVD	Bhattacharyya	MBDL	FA	t-Score	ICA	MRMRe	NMF	ANOVA	None	Kendall	LASSO	RFE-LR	ET	Boruta
Boruta	-26 (-0.038)	-16 (-0.035)	-16 (-0.025)	-17 (-0.021)	-14 (-0.02)	-12 (-0.022)	-14 (-0.019)	-5 (-0.018)	-11 (-0.012)	-8 (-0.02)	-13 (-0.013)	-6 (-0.018)	-9 (-0.012)	-3 (-0.013)	-7 (-0.009)	-2 (-0.003)	-3 (-0.004)	-9 (-0.004)	
ET	-23 (-0.033)	-23 (-0.031)	-18 (-0.021)	-17 (-0.016)	-13 (-0.016)	-12 (-0.018)	-19 (-0.015)	-6 (-0.013)	-6 (-0.008)	-8 (-0.016)	-4 (-0.009)	-1 (-0.013)	-6 (-0.008)	-4 (-0.009)	1 (-0.005)	-10 (0.002)	-4 (0.0)		9 (0.004)
RFE-LR	-27 (-0.034)	-18 (-0.031)	-18 (-0.021)	-14 (-0.017)	-19 (-0.016)	-11 (-0.018)	-10 (-0.015)	-9 (-0.014)	-14 (-0.008)	-4 (-0.016)	-12 (-0.009)	-3 (-0.014)	-9 (-0.008)	0 (-0.009)	-6 (-0.005)	2 (0.001)		4 (-0.0)	3 (0.004)
LASSO	-28 (-0.035)	-18 (-0.033)	-16 (-0.022)	-13 (-0.018)	-16 (-0.018)	-14 (-0.02)	-12 (-0.017)	-10 (-0.015)	-9 (-0.009)	-8 (-0.018)	-9 (-0.011)	-7 (-0.015)	-1 (-0.01)	-3 (-0.011)	4 (-0.007)		-2 (-0.001)	10 (-0.002)	2 (0.003)
Kendall	-22 (-0.028)	-13 (-0.026)	-12 (-0.016)	-8 (-0.011)	-9 (-0.011)	-5 (-0.013)	-10 (-0.01)	-9 (-0.008)	-12 (-0.003)	-8 (-0.011)	-2 (-0.004)	2 (-0.008)	0 (-0.003)	-7 (-0.004)		-4 (0.007)	6 (0.005)	-1 (0.005)	7 (0.009)
None	-18 (-0.024)	-16 (-0.022)	-14 (-0.012)	-11 (-0.007)	-8 (-0.007)	-5 (-0.009)	-6 (-0.006)	1 (-0.004)	-6 (0.001)	-3 (-0.007)	5 (0.0)	0 (-0.004)	-4 (0.001)		7 (0.004)	3 (0.011)	0 (0.009)	4 (0.009)	3 (0.013)
ANOVA	-20 (-0.026)	-14 (-0.023)	-6 (-0.013)	-11 (-0.009)	-9 (-0.008)	-6 (-0.01)	-12 (-0.007)	-7 (-0.006)	5 (0.0)	-5 (-0.008)	-2 (-0.001)	-6 (-0.005)		4 (-0.001)	0 (0.003)	1 (0.01)	9 (0.008)	6 (0.008)	9 (0.012)
NMF	-25 (-0.02)	-17 (-0.018)	-10 (-0.007)	-11 (-0.003)	-1 (-0.003)	-7 (-0.005)	2 (-0.002)	-5 (-0.0)	-1 (0.006)	5 (-0.003)	0 (0.004)		6 (0.005)	0 (0.004)	-2 (0.008)	7 (0.015)	3 (0.014)	1 (0.013)	6 (0.018)
MRMRe	-14 (-0.024)	-6 (-0.022)	-7 (-0.012)	-9 (-0.007)	-8 (-0.007)	-3 (-0.009)	-5 (-0.006)	-4 (-0.004)	3 (0.001)	-4 (-0.007)		0 (-0.004)	2 (0.001)	-5 (-0.0)	2 (0.004)	9 (0.011)	12 (0.009)	4 (0.009)	13 (0.013)
ICA	-18 (-0.017)	-13 (-0.015)	-4 (-0.005)	-12 (-0.0)	-12 (0.0)	-5 (-0.002)	-3 (0.001)	0 (0.003)	0 (0.008)		4 (0.007)	-5 (0.003)	5 (0.008)	3 (0.007)	8 (0.011)	8 (0.018)	4 (0.016)	8 (0.016)	8 (0.02)
t-Score	-15 (-0.026)	-12 (-0.023)	-9 (-0.013)	-7 (-0.009)	-5 (-0.008)	-7 (-0.01)	-7 (-0.007)	0 (-0.006)		0 (-0.008)	-3 (-0.001)	1 (-0.006)	-5 (-0.0)	6 (-0.001)	12 (0.003)	9 (0.009)	14 (0.008)	6 (0.008)	11 (0.012)
FA	-8 (-0.02)	-8 (-0.018)	-6 (-0.007)	-8 (-0.003)	-6 (-0.002)	-3 (-0.005)	4 (-0.002)		0 (0.006)	0 (-0.003)	4 (0.004)	5 (0.0)	7 (0.006)	-1 (0.004)	9 (0.008)	10 (0.015)	9 (0.014)	6 (0.013)	5 (0.018)
MBDL	-23 (-0.019)	-12 (-0.016)	-11 (-0.006)	-6 (-0.002)	-1 (-0.001)	0 (-0.003)		-4 (0.002)	7 (0.007)	3 (-0.001)	5 (0.006)	-2 (0.002)	12 (0.007)	6 (0.006)	10 (0.01)	12 (0.017)	10 (0.015)	19 (0.015)	14 (0.019)
Bhattacharyya	-12 (-0.015)	-13 (-0.013)	-1 (-0.003)	-3 (0.002)	-4 (0.002)		0 (0.003)	3 (0.005)	7 (0.01)	5 (0.002)	3 (0.009)	7 (0.005)	6 (0.01)	5 (0.009)	5 (0.013)	14 (0.02)	11 (0.018)	12 (0.018)	12 (0.022)
TruncatedSVD	-9 (-0.018)	-11 (-0.015)	-6 (-0.005)	0 (-0.001)		4 (-0.002)	1 (0.001)	6 (0.002)	5 (0.008)	12 (-0.0)	8 (0.007)	1 (0.003)	9 (0.008)	8 (0.007)	9 (0.011)	16 (0.018)	19 (0.016)	13 (0.016)	14 (0.02)
PCA	-9 (-0.017)	-12 (-0.015)	-10 (-0.004)		0 (0.001)	3 (-0.002)	6 (0.002)	8 (0.003)	7 (0.009)	12 (0.0)	9 (0.007)	11 (0.003)	11 (0.009)	11 (0.007)	8 (0.011)	13 (0.018)	14 (0.017)	17 (0.016)	17 (0.021)
kPCA	-12 (-0.013)	-8 (-0.01)		10 (0.004)	6 (0.005)	1 (0.003)	11 (0.006)	6 (0.007)	9 (0.013)	4 (0.005)	7 (0.012)	10 (0.007)	6 (0.013)	14 (0.012)	12 (0.016)	16 (0.022)	18 (0.021)	18 (0.021)	16 (0.025)
UMAP	-4 (-0.002)		8 (0.01)	12 (0.015)	11 (0.015)	13 (0.013)	12 (0.016)	8 (0.018)	12 (0.023)	13 (0.015)	6 (0.022)	17 (0.018)	14 (0.023)	16 (0.022)	13 (0.026)	18 (0.033)	18 (0.031)	23 (0.031)	16 (0.035)
SRP		4 (0.002)	12 (0.013)	9 (0.017)	9 (0.018)	12 (0.015)	23 (0.019)	8 (0.02)	15 (0.026)	18 (0.017)	14 (0.024)	25 (0.02)	20 (0.026)	18 (0.024)	22 (0.028)	28 (0.035)	27 (0.034)	23 (0.033)	26 (0.038)

Figure S3C Benefit and gain comparison in F0.5 score

The top number in each cell indicates the number of datasets, where replacing one method with another method results in a gain or loss in performance. The number in parentheses represents the average gain in this case.

Benefit and gain comparisons in F2 score

	SRP	PCA	UMAP	None	MBDL	TruncatedSVD	Bhattacharyya	ICA	FA	kPCA	Kendall	RFE-LR	ANOVA	t-Score	ET	Boruta	MRMRe	NMF	LASSO
LASSO	-26 (-0.011)	-16 (-0.005)	-19 (-0.005)	-16 (-0.005)	-16 (-0.005)	-8 (-0.006)	-10 (-0.006)	-6 (-0.005)	-10 (-0.005)	-11 (-0.005)	-8 (-0.001)	-4 (-0.0)	-2 (-0.002)	-8 (-0.001)	-2 (-0.001)	0 (-0.001)	-2 (-0.0)	-2 (-0.003)	
NMF	-24 (-0.008)	-16 (-0.002)	-19 (-0.002)	-13 (-0.002)	-11 (-0.002)	-16 (-0.003)	-11 (-0.003)	-11 (-0.002)	-6 (-0.002)	-6 (-0.002)	-10 (0.002)	0 (0.003)	-4 (0.001)	-6 (0.002)	2 (0.002)	9 (0.002)	3 (0.003)		2 (0.003)
MRMRe	-26 (-0.011)	-16 (-0.005)	-10 (-0.005)	-14 (-0.004)	-12 (-0.005)	-18 (-0.005)	0 (-0.006)	-10 (-0.005)	-4 (-0.005)	-5 (-0.004)	-6 (-0.001)	-5 (-0.0)	-10 (-0.001)	0 (-0.0)	3 (-0.0)	0 (-0.0)		-3 (-0.003)	2 (0.0)
Boruta	-23 (-0.011)	-18 (-0.004)	-5 (-0.004)	-16 (-0.004)	-11 (-0.005)	-17 (-0.005)	-6 (-0.005)	-7 (-0.004)	-6 (-0.005)	-10 (-0.004)	5 (-0.001)	-9 (0.0)	6 (-0.001)	2 (0.0)	-2 (0.0)		0 (0.0)	-9 (-0.002)	0 (0.001)
ET	-22 (-0.011)	-13 (-0.005)	-14 (-0.005)	-5 (-0.004)	-14 (-0.005)	-5 (-0.005)	-4 (-0.005)	-5 (-0.005)	-8 (-0.005)	-5 (-0.004)	-4 (-0.001)	-1 (0.0)	2 (-0.001)	-1 (0.0)		2 (-0.0)	-3 (0.0)	-2 (-0.002)	2 (0.001)
t-Score	-18 (-0.011)	-9 (-0.005)	-12 (-0.005)	-9 (-0.004)	-10 (-0.005)	-3 (-0.005)	-2 (-0.005)	-4 (-0.005)	-6 (-0.005)	-5 (-0.004)	-9 (-0.001)	-6 (0.0)	1 (-0.001)		1 (-0.0)	-2 (-0.0)	0 (0.0)	6 (-0.002)	8 (0.001)
ANOVA	-20 (-0.009)	-13 (-0.003)	-5 (-0.003)	-7 (-0.003)	-8 (-0.004)	-9 (-0.004)	-2 (-0.004)	-2 (-0.003)	3 (-0.004)	-4 (-0.003)	-6 (0.0)	-1 (0.001)		-1 (0.001)	-2 (0.001)	-6 (0.001)	10 (0.001)	4 (-0.001)	2 (0.002)
RFE-LR	-19 (-0.011)	-8 (-0.005)	-11 (-0.005)	-8 (-0.004)	-5 (-0.005)	-9 (-0.005)	-6 (-0.006)	0 (-0.005)	-3 (-0.005)	-1 (-0.004)	1 (-0.001)		1 (-0.001)	6 (-0.0)	1 (-0.0)	9 (-0.0)	5 (0.0)	0 (-0.003)	4 (0.0)
Kendall	-25 (-0.01)	-7 (-0.004)	-11 (-0.004)	-8 (-0.003)	-12 (-0.004)	0 (-0.004)	-3 (-0.005)	-1 (-0.004)	-6 (-0.004)	2 (-0.003)		-1 (0.001)	6 (-0.0)	9 (0.001)	4 (0.001)	-5 (0.001)	6 (0.001)	10 (-0.002)	8 (0.001)
kPCA	-17 (-0.006)	-1 (-0.0)	-9 (-0.0)	-2 (0.0)	-2 (-0.001)	6 (-0.001)	-5 (-0.001)	-6 (-0.0)	-3 (-0.001)		-2 (0.003)	1 (0.004)	4 (0.003)	5 (0.004)	5 (0.004)	10 (0.004)	5 (0.004)	6 (0.002)	11 (0.005)
FA	-13 (-0.006)	-7 (0.0)	-7 (0.0)	-7 (0.001)	-4 (-0.0)	2 (-0.0)	1 (-0.001)	0 (0.0)		3 (0.001)	6 (0.004)	3 (0.005)	-3 (0.004)	6 (0.005)	8 (0.005)	6 (0.005)	4 (0.005)	6 (0.002)	10 (0.005)
ICA	-14 (-0.006)	-6 (0.0)	-4 (-0.0)	-4 (0.0)	1 (-0.0)	5 (-0.0)	-3 (-0.001)		0 (-0.0)	6 (0.0)	1 (0.004)	0 (0.005)	2 (0.003)	4 (0.005)	5 (0.005)	7 (0.004)	10 (0.005)	11 (0.002)	6 (0.005)
Bhattacharyya	-19 (-0.005)	-4 (0.001)	-3 (0.001)	-1 (0.001)	0 (0.001)	5 (0.0)		3 (0.001)	-1 (0.001)	5 (0.001)	3 (0.005)	6 (0.006)	2 (0.004)	2 (0.005)	4 (0.005)	6 (0.005)	0 (0.006)	11 (0.003)	10 (0.006)
TruncatedSVD	-12 (-0.006)	-7 (0.001)	-3 (0.0)	0 (0.001)	4 (0.0)		-5 (-0.0)	-5 (0.0)	-2 (0.0)	-6 (0.001)	0 (0.004)	9 (0.005)	9 (0.004)	3 (0.005)	5 (0.005)	17 (0.005)	18 (0.005)	16 (0.003)	8 (0.006)
MBDL	-11 (-0.006)	-9 (0.0)	-2 (0.0)	5 (0.001)		-4 (-0.0)	0 (-0.001)	-1 (0.0)	4 (0.0)	2 (0.001)	12 (0.004)	5 (0.005)	8 (0.004)	10 (0.005)	14 (0.005)	11 (0.005)	12 (0.005)	11 (0.002)	16 (0.005)
None	-9 (-0.007)	0 (-0.0)	-7 (-0.0)		-5 (-0.001)	0 (-0.001)	1 (-0.001)	4 (-0.0)	7 (-0.001)	2 (-0.0)	8 (0.003)	8 (0.004)	7 (0.003)	9 (0.004)	5 (0.004)	16 (0.004)	14 (0.004)	13 (0.002)	16 (0.005)
UMAP	-15 (-0.006)	1 (0.0)		7 (0.0)	2 (-0.0)	3 (-0.0)	3 (-0.001)	4 (0.0)	7 (-0.0)	9 (0.0)	11 (0.004)	11 (0.005)	5 (0.003)	12 (0.005)	14 (0.005)	5 (0.004)	10 (0.005)	19 (0.002)	19 (0.005)
PCA	-8 (-0.006)		-1 (-0.0)	0 (0.0)	9 (-0.0)	7 (-0.001)	4 (-0.001)	6 (-0.0)	7 (-0.0)	1 (0.0)	7 (0.004)	8 (0.005)	13 (0.003)	9 (0.005)	13 (0.005)	18 (0.004)	16 (0.005)	16 (0.002)	16 (0.005)
SRP		8 (0.006)	15 (0.006)	9 (0.007)	11 (0.006)	12 (0.006)	19 (0.005)	14 (0.006)	13 (0.006)	17 (0.006)	25 (0.01)	19 (0.011)	20 (0.009)	18 (0.011)	22 (0.011)	23 (0.011)	26 (0.011)	24 (0.008)	26 (0.011)

Figure S3D Benefit and gain comparison in F2 score

The top number in each cell indicates the number of datasets, where replacing one method with another method results in a gain or loss in performance. The number in parentheses represents the average gain in this case.

Relationship regarding dataset characteristics

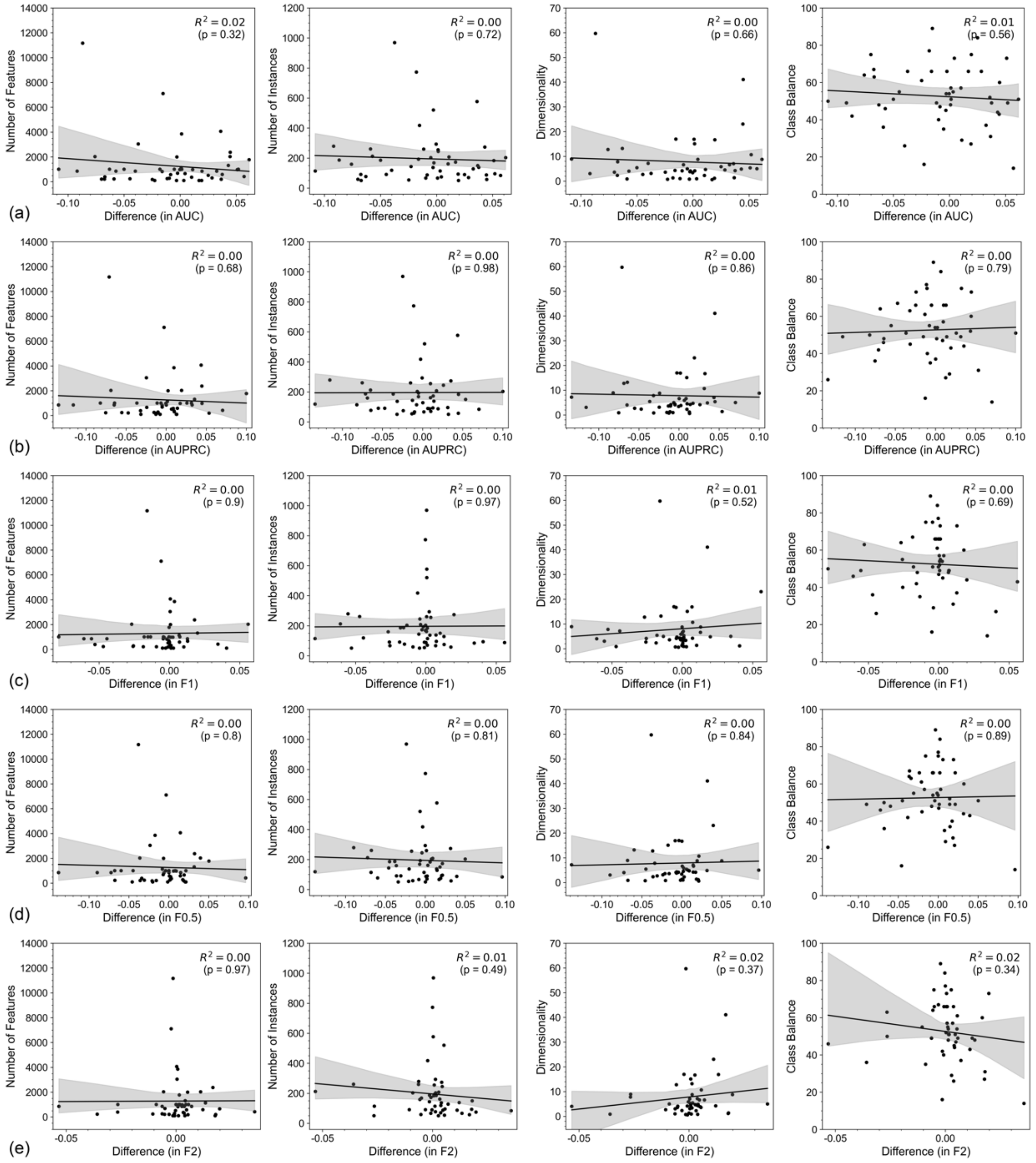

Figure S4 Relationship between the observed difference and dataset characteristics.

Each scatter plot displays a dataset characteristic against the difference in (a) AUC, (b) AUPRC, (c) F1 score, (d) F0.5 score, and (e) F2 score between the best feature projection and selection methods. From left to right: number of features, number of instances, dimensionality, and balance. The lines represent a linear regression of the corresponding variables. The R^2 and p-values for the linear regression are shown in each subplot. The shaded areas represent the 95% confidence intervals for the regression predictions obtained by bootstrapping.

Computational efficiency

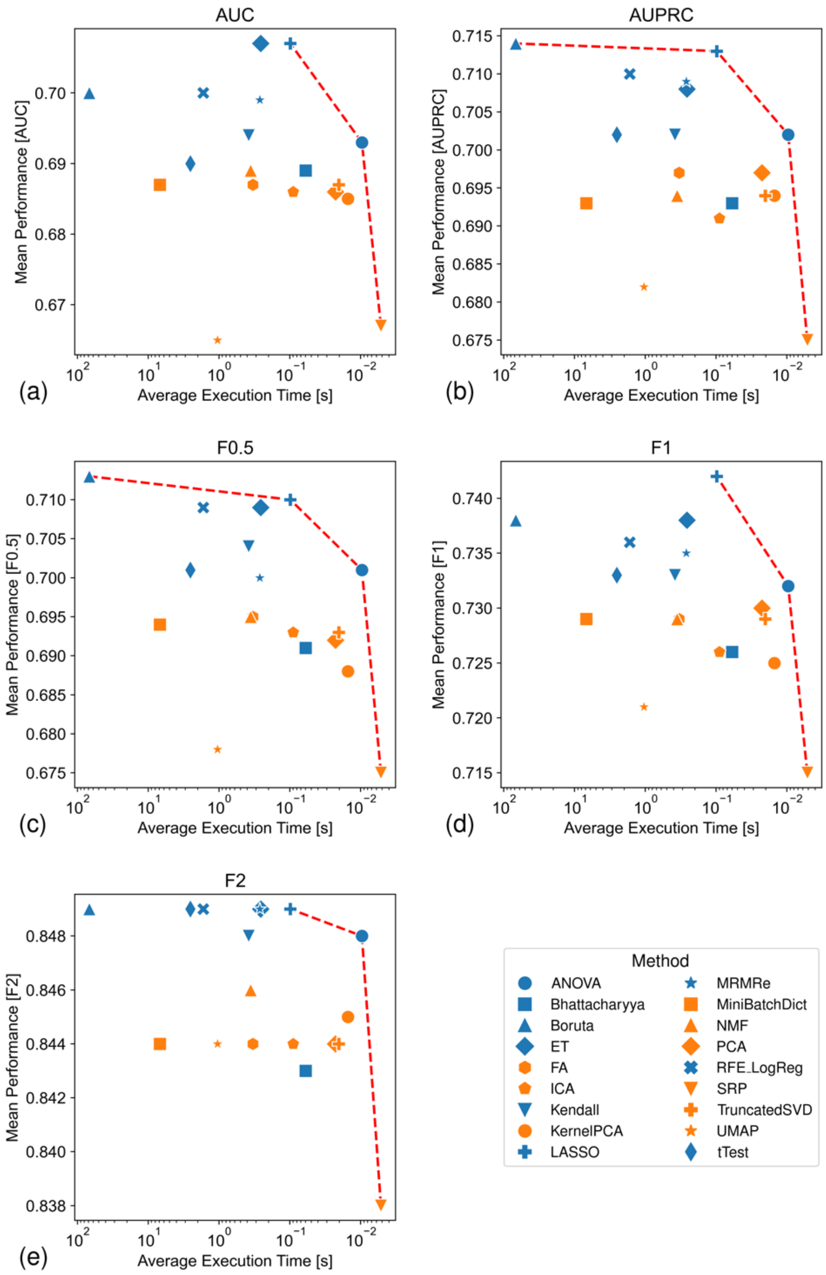

Figure S5 Computational efficiency
Each plot shows the average computation time (in seconds on a logarithmic scale) versus average performance across all datasets for each method. The red line indicates the Pareto front, comprising methods that are not outperformed in both speed and performance. Metrics shown: (a) AUC, (b) AUPRC, (c) F0.5-score, (d) F1-score, (e) F2-score.

Execution time

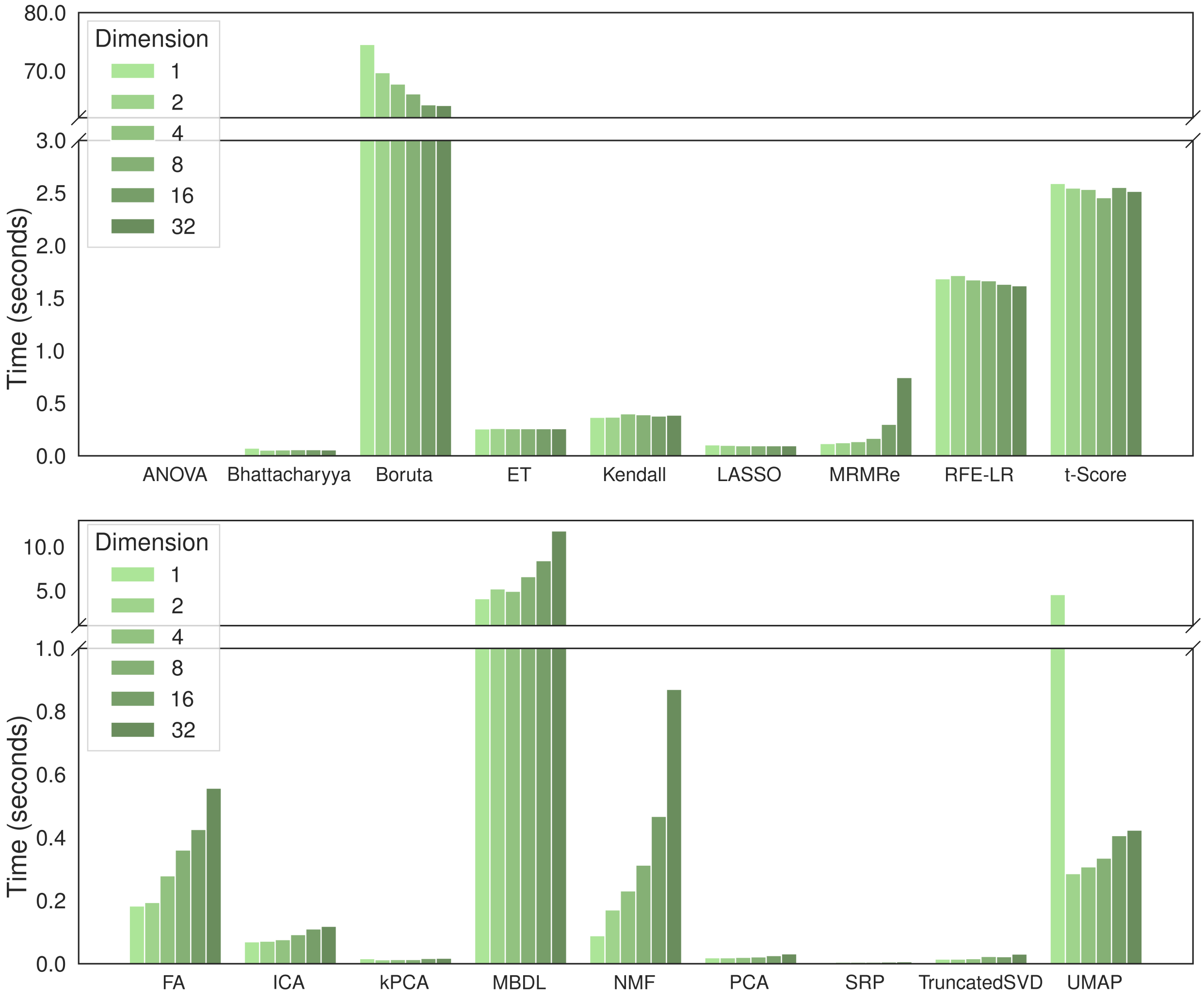

Figure S6 Execution time of the methods.
The plot shows the time (in seconds) required by each method for different values N of reduced features. The top subplot shows the feature selection methods, and the bottom subplot shows the projection methods.

AUC results

Table S1 Comparison of AUC between projection and selection methods across all datasets.

Dataset	Projection	AUC of projection method	Selection	AUC of selection method	Difference in AUC
WORC-Lipo	PCA	0.758 +/- 0.028	Boruta	0.866 +/- 0.017	-0.108
Dong2022	ICA	0.712 +/- 0.021	Boruta	0.804 +/- 0.018	-0.092
UPENN-GBM	KernelPCA	0.498 +/- 0.025	Bhattacharyya	0.585 +/- 0.028	-0.087
LGG-1p19qDeletion	KernelPCA	0.768 +/- 0.038	Boruta	0.844 +/- 0.016	-0.076
QIN-HEADNECK	SRP	0.476 +/- 0.061	ANOVA	0.546 +/- 0.066	-0.070
Head-Neck-PET-CT	PCA	0.701 +/- 0.047	ANOVA	0.769 +/- 0.026	-0.068
Li2020	NMF	0.735 +/- 0.043	Bhattacharyya	0.802 +/- 0.034	-0.067
WORC-CRLM	SRP	0.567 +/- 0.040	MRMRe	0.630 +/- 0.062	-0.063
Deng2023	FA	0.786 +/- 0.019	RFE-LR	0.845 +/- 0.017	-0.059
Huang2023	ICA	0.902 +/- 0.020	MRMRe	0.959 +/- 0.007	-0.057
WORC-Liver	MBDL	0.663 +/- 0.039	Bhattacharyya	0.713 +/- 0.031	-0.050
Ramella2018	MBDL	0.615 +/- 0.059	RFE-LR	0.660 +/- 0.055	-0.045
Dai2023	KernelPCA	0.720 +/- 0.023	Bhattacharyya	0.760 +/- 0.033	-0.040
PI-CAI	ICA	0.708 +/- 0.010	ET	0.745 +/- 0.009	-0.037
Fusco2022	FA	0.657 +/- 0.048	Kendall	0.681 +/- 0.043	-0.025
NSCLC-Radiogenomics	FA	0.577 +/- 0.040	LASSO	0.600 +/- 0.046	-0.023
Prostate-MRI-US-Biopsy	FA	0.682 +/- 0.013	Bhattacharyya	0.700 +/- 0.009	-0.018
Zhang2024B	FA	0.626 +/- 0.023	ET	0.642 +/- 0.028	-0.016
UCSF-PDGM	PCA	0.893 +/- 0.018	ET	0.909 +/- 0.015	-0.015
Song2020	KernelPCA	0.950 +/- 0.004	ANOVA	0.962 +/- 0.010	-0.012
Wang2024	SRP	0.669 +/- 0.051	Boruta	0.679 +/- 0.046	-0.010
Granata2021	UMAP	0.628 +/- 0.065	MRMRe	0.637 +/- 0.035	-0.009
WORC-Desmoid	FA	0.799 +/- 0.034	LASSO	0.804 +/- 0.020	-0.005
Head-Neck-Radiomics-HN1	SRP	0.733 +/- 0.018	tTest	0.737 +/- 0.020	-0.003
Hunter2023	NMF	0.817 +/- 0.008	ANOVA	0.820 +/- 0.012	-0.003
Arita2018	PCA	0.755 +/- 0.025	ET	0.758 +/- 0.025	-0.002
Hosny2018A	NMF	0.564 +/- 0.025	MRMRe	0.564 +/- 0.029	-0.000
OcanaTienda2023	UMAP	0.495 +/- 0.065	ET	0.494 +/- 0.078	0.001
Zhang2024A	SRP	0.716 +/- 0.003	Bhattacharyya	0.715 +/- 0.016	0.001
WORC-GIST	NMF	0.709 +/- 0.021	MRMRe	0.707 +/- 0.021	0.001
Ahn2021	KernelPCA	0.507 +/- 0.045	Kendall	0.504 +/- 0.039	0.003
Lu2019	UMAP	0.678 +/- 0.051	Boruta	0.674 +/- 0.044	0.004
ISPY1	SRP	0.559 +/- 0.041	RFE-LR	0.549 +/- 0.022	0.010
Hosny2018B	SRP	0.657 +/- 0.013	LASSO	0.646 +/- 0.037	0.011
LNDb	NMF	0.814 +/- 0.024	ET	0.797 +/- 0.022	0.017

HNSCC	UMAP	0.754 +/- 0.050	RFE-LR	0.735 +/- 0.048	0.019
Granata2024	PCA	0.653 +/- 0.043	ANOVA	0.633 +/- 0.079	0.019
Colorectal-Liver-Metastases	FA	0.604 +/- 0.081	tTest	0.579 +/- 0.054	0.025
C4KC-KiTS	FA	0.597 +/- 0.049	LASSO	0.569 +/- 0.036	0.028
Petrillo2023	KernelPCA	0.525 +/- 0.060	MRMRe	0.492 +/- 0.039	0.033
BraTS-2021	ICA	0.603 +/- 0.017	LASSO	0.567 +/- 0.016	0.036
Veeraraghavan2020	NMF	0.549 +/- 0.040	LASSO	0.512 +/- 0.037	0.037
Sasaki2019	NMF	0.570 +/- 0.046	Bhattacharyya	0.532 +/- 0.052	0.038
Keek2020	UMAP	0.667 +/- 0.015	tTest	0.624 +/- 0.022	0.043
Meningioma-SEG-CLASS	SRP	0.662 +/- 0.018	LASSO	0.617 +/- 0.054	0.044
Brancato2023	TruncatedSVD	0.763 +/- 0.038	Boruta	0.718 +/- 0.036	0.045
Hosny2018C	ICA	0.568 +/- 0.038	Bhattacharyya	0.517 +/- 0.033	0.051
WORC-Melanoma	KernelPCA	0.556 +/- 0.036	RFE-LR	0.504 +/- 0.040	0.052
HCC-TACE-Seg	FA	0.552 +/- 0.079	tTest	0.495 +/- 0.062	0.057
Zhang2023	KernelPCA	0.652 +/- 0.018	Bhattacharyya	0.590 +/- 0.024	0.061

For each dataset, the best-performing projection and selection methods are indicated along with their corresponding AUC values. The final column shows the difference in AUC between the projection and selection methods.

References

1. Jolliffe, ian. *Principal Component Analysis*. (Springer-Verlag, New York, 2002). doi:10.1007/b98835.
2. Mika, S. *et al.* Kernel PCA and De-Noising in Feature Spaces. in *Advances in Neural Information Processing Systems* vol. 11 (MIT Press, 1998).
3. Schölkopf, B. The Kernel Trick for Distances. in *Advances in Neural Information Processing Systems* vol. 13 (MIT Press, 2000).
4. Comon, P. Independent component analysis, A new concept? *Signal Process.* **36**, 287–314 (1994).

5. Bartholomew, D., Knott, M. & Moustaki, I. *Latent Variable Models and Factor Analysis: A Unified Approach*. (Wiley, 2011). doi:10.1002/9781119970583.
6. Wang, Y.-X. & Zhang, Y.-J. Nonnegative Matrix Factorization: A Comprehensive Review. *IEEE Trans. Knowl. Data Eng.* **25**, 1336–1353 (2013).
7. Liu, W., Zheng, N. & You, Q. Nonnegative matrix factorization and its applications in pattern recognition. *Chin. Sci. Bull.* **51**, 7–18 (2006).
8. Mairal, J., Bach, F., Ponce, J. & Sapiro, G. Online dictionary learning for sparse coding. in *Proceedings of the 26th Annual International Conference on Machine Learning* 689–696 (ACM, Montreal Quebec Canada, 2009). doi:10.1145/1553374.1553463.
9. Falini, A. A review on the selection criteria for the truncated SVD in Data Science applications. *J. Comput. Math. Data Sci.* **5**, 100064 (2022).
10. McInnes, L., Healy, J. & Melville, J. UMAP: Uniform Manifold Approximation and Projection for Dimension Reduction. Preprint at https://doi.org/10.48550/arXiv.1802.03426 (2020).
11. Bair, E., Hastie, T., Paul, D. & Tibshirani, R. Prediction by Supervised Principal Components. *J. Am. Stat. Assoc.* **101**, 119–137 (2006).
12. Karimi, A.-H., Wong, A. & Ghodsi, A. SRP: Efficient class-aware embedding learning for large-scale data via supervised random projections. Preprint at https://doi.org/10.48550/arXiv.1811.03166 (2018).
13. Tibshirani, R. Regression Shrinkage and Selection via the Lasso. *J. R. Stat. Soc. Ser. B Methodol.* **58**, 267–288 (1996).
14. Ding, C. & Peng, H. Minimum redundancy feature selection from microarray gene expression data. *J. Bioinform. Comput. Biol.* **03**, 185–205 (2005).
15. De Jay, N. *et al.* mRMRe: an R package for parallelized mRMR ensemble feature selection. *Bioinformatics* **29**, 2365–2368 (2013).
16. Kailath, T. The Divergence and Bhattacharyya Distance Measures in Signal Selection. *IEEE Trans. Commun. Technol.* **15**, 52–60 (1967).

17. Guorong, X., Peiqi, C. & Minhui, W. Bhattacharyya distance feature selection. in *Proceedings of 13th International Conference on Pattern Recognition* vol. 2 195–199 vol.2 (1996).

18. Kendall, M. G. A new measure of rank correlation. *Biometrika* **30**, 81–93 (1938).

19. Chattamvelli, R. Rank Correlation. in *Correlation in Engineering and the Applied Sciences* 77–106 (Springer Nature Switzerland, Cham, 2024). doi:10.1007/978-3-031-51015-1_3.

20. Guyon, I., Weston, J., Barnhill, S. & Vapnik, V. Gene Selection for Cancer Classification using Support Vector Machines. *Mach. Learn.* **46**, 389–422 (2002).

21. Kursa, M. B., Jankowski, A. & Rudnicki, W. R. Boruta – A System for Feature Selection. *Fundam. Informaticae* **101**, 271–285 (2010).

22. Geurts, P., Ernst, D. & Wehenkel, L. Extremely randomized trees. *Mach. Learn.* **63**, 3–42 (2006).
